# Efficacy of acupuncture combined with mirror therapy in the treatment of post-stroke limb movement disorders: a systematic review and meta-analysis of randomised controlled trials

**DOI:** 10.3389/fresc.2024.1464502

**Published:** 2024-11-07

**Authors:** Weihao Ke, Hongxin Cheng, Xiaoxuan Ren, Liang Yang, Xiaomin Lai, Zhenyu Wang

**Affiliations:** ^1^ Department of Rehabilitation Medicine, The Affiliated Yong Chuan Hospital of Chong Qing Medical University, Chongqing, China; ^2^ Sichuan Province Orthopaedic Hospital, Department of Neck, Shoulder, Back, and Leg Pain, Wuhou District, Chengdu, China; ^3^ The Affiliated Rehabilitation Hospital of Chong Qing Medical University, Administrative Department, Jiulongpo District, Chongqing, China

**Keywords:** acupuncture, mirror therapy, stroke, motor impairment, meta-analysis

## Abstract

**Objective:**

To investigate whether the combination of acupuncture and mirror therapy can improve motor impairment in stroke patients.

**Design:**

A systematic review and meta-analysis of randomised controlled trials.

**Data sources:**

CNKI, Wanfang, PubMed, Embase, Vip, web of since, Cochrane database and CBM database.

**Eligibility criteria for selecting studies:**

The included randomized controlled trials compared the efficacy of acupuncture therapy (AT) combined with mirror therapy (MT) against AT, MT, and conventional rehabilitation therapy on limb motor impairment in stroke patients, with independent data extraction and study quality assessment conducted. A META analysis using fixed-effect and random-effect models was performed to calculate the mean difference (MD) in motor scores and the Total effective rate RR (Risk ratio) between the AT combined with MT group and the control group.

**Main outcome measures:**

The Fugl-Meyer Assessment (FMA) for motor function includes the FMA-T (total FMA), FMA-UE (upper extremity FMA), and FMA-L (lower extremity FMA).

**Results:**

A total of 42 randomized controlled trials were included, involving 3,340 patients with post-stroke motor impairment. AT combined with MT was more favorable for FMA-UE (mean difference [MD] = 6.67, 95% CI [5.60–7.93], Z = 11.42, *P* < 0.0001), FMA-L [MD = 3.37, 95% CI (2.99–3.76), Z = 17.31, *P* < 0.001], and FMA-T [MD = 6.84, 95% CI (5.92–7.77), Z = 14.48, *P* < 0.001]. The combined AT and MT treatment was more favorable for the Modified Barthel Index (MBI) score in post-stroke motor impairment [MD = 10.82, 95% CI (8.52–13.12), Z = 9.22, *P* < 0.001]. AT combined with MT was more favorable for the Modified Ashworth Scale (MAS) [MD = −0.34, 95% CI (−0.66 to −0.03), Z = 14.48, *P* < 0.001]. AT combined with MT was more favorable for the Total effective rate in treating post-stroke motor impairment (relative risk = 1.27, 95% confidence interval [CI] [1.19–1.37], Z = 6.54, *P* < 0.001).

**Conclusions:**

AT combined with MT can effectively improve patients’ motor function and daily living abilities.

**Systematic Review Registration:**

PROSPERO, identifier, CRD42024559992.

## Introduction

Stroke is a globally prevalent and very dangerous disease, with more than 15 million new cases each year. More than 60% of stroke patients face varying degrees of physical disability after the onset of the disease. When cardiovascular disease is considered alone, stroke is the fifth most lethal disease after heart disease, cancer, COVID-19, and unintentional injuries (1). As an outcome of stroke, numerous patients endure long-term motor deficits that can range from unilateral to bilateral paralysis (2–4). Additionally, it can significantly impair their cognitive, memory, speech, respiration, vision, and motor functions. The daily quality of life of stroke victims is significantly impacted by the loss of limb movement ability. Consequently, it is imperative to implement a vigorous approach to the treatment of limb movement disorders in order to facilitate the reintegration of patients into a typical lifestyle and alleviate the burden on their families.

Mostly, traditional non-invasive stroke treatments consist of physiotherapy and pharmacological therapies. Traditional physiotherapy techniques include active and passive movement therapy, balance and gait training, fine motor skill development, physical factor therapy, and occupational therapy (5, 6). Pharmacological treatments often involve anticoagulants, antiplatelet agents, lipid-lowering drugs, and medications that enhance microcirculation (7). However, traditional physiotherapy methods often show limited effectiveness, and pharmacological treatments may cause various side effects. Given these limitations, there is an urgent need to explore innovative physiotherapy approaches to improve treatment efficacy and the quality of life for stroke patients.

Commonly used non-invasive stroke treatment options include physiotherapy and medicines. The physiotherapy interventions mentioned here, although not universally standardized, include active and passive movement therapy, balance and gait training, fine motor skill development, physical factor therapy, and occupational therapy (5, 6). These usually include anticoagulants, antiplatelet medicines, lipid-lowering medications, and pharmaceuticals that increase microcirculation (7). However, these traditional physical therapy methods usually have limited effectiveness. In addition, pharmacological treatments may lead to various side effects. In view of these limitations, there is an urgent need to explore innovative physiotherapy approaches to enhance treatment efficacy and improve the quality of life of stroke patients.

Acupuncture therapy (AT) is a traditional Chinese medicine treatment that works by stimulating specific points on the body to improve the flow of energy and blood. This stimulation helps increase blood circulation to the brain and supports the brain's ability to recover and adapt by promoting changes in its structure and function (known as neuroplasticity). The World Health Organization (WHO) recognizes the importance of AT in stroke rehabilitation (8, 9). In addition, the National Institute for Health and Clinical Excellence (UK) has acknowledged mirror therapy (MT) as a supplementary treatment for post-stroke movement problems (6, 10, 11). Recent studies (12) have emphasized the notable impact of integrating AT treatment with MT. However, current systematic review (13) have certain drawbacks, notably in regards to subgroup analyses of specific AT techniques, treatment length, and disease stage. The objective of this meta-analysis was to methodically evaluate the impact of integrating AT with MT on movement impairments following a stroke. The purpose was to enhance and improve rehabilitation treatment approaches in clinical practice.

## Methods

### Protocol and guidance

The Reporting Specification for Systematic Evaluation/Meta-Analysis (PRISMA) (14) guided the conduct of this study, and PROSPERO has registered the protocol for this review (CRD42024559992).

### Inclusion criteria

We considered trials to be eligible. If trials enrolled stroke patients with movement disorders (age ≥18) diagnosed according to any recognized diagnostic criteria, if there were any randomized controlled trials or clinical trials evaluating the efficacy of AT combined with MT for patients diagnosed with upper or lower limb dysfunction, if the studies included at least one of the following parameters as outcome variables: limb motor ability, degree of muscle spasm, clinical efficacy, and ability in daily life activities; if the intervention was AT + MT and the control group was monotherapy, such as AT or MT or conventional rehabilitation.

### Exclusion criteria

We excluded studies if they were case reports, *in vivo* animal studies, reviews, or interviews; if they were registered without any outcome data or if data were missing and we were unable to contact the original authors to obtain the relevant outcome data; if they were randomized controlled trials assessing only the efficacy of AT alone or MT alone in stroke patients; if the study content did not have relevant outcome variables.

### Outcomes

The primary outcome measures were FMA-T, FMA-UE, and FMA-L. Modified MAS, MBI, and Total effective rate served as the secondary outcome measures. Supplementary eTable S1 shows the definitions of these outcomes.

### Search strategy

One of the authors (XX Ren) searched the following databases: the CNKI, Wanfang, PubMed, Embase, Vip, Web of Since, and the Cochrane database and CBM database. We did not restrict the language. The search was conducted from the beginning until July 3, 2024. Supplementary eTable S2 describes the search strategy.

### Study selection

After removing duplicates, two independent researchers (L Y and XM Lai) screened all titles and abstracts. They were provided with the full text and carried out further screening when deemed eligible.

### Data collection process

Two independent researchers (L Y and XM Lai) extracted data from the included trials using standard data extraction forms. If a randomized controlled trial had more than two treatment groups, we pooled data from the different treatment groups. If a study mentioned an outcome of interest but did not provide an estimate, we contacted the authors to obtain the data. If there were disagreements, we resolved them by consensus.

### Assessment of risk of bias and quality of evidence

The quality of eligible studies was independently assessed by two reviewers (HX Chen, WH Ke) using the Cochrane Collaboration Risk of Bias Tool (15, 16), and in case of disagreement, it was discussed and resolved with a third reviewer (XX Ren).

### Data synthesis

Meta-analysis was performed using RevMan 5.3 and Stata 16.0 software. Mean difference (MD) was used as the effect size (effect size) for continuous outcome indicators, and RR (risk ratio) was used as the effect size for dichotomous variables. The Q test and I statistic were used to examine inter-study heterogeneity; if *P* > 0.1 or I^2^ < 50%, it meant that there was no heterogeneity between studies and a fixed-effects combined effect size was needed, and vice versa, a random-effects combined effect size was used. Further, the possibility of publication bias was assessed qualitatively by visual estimation of the funnel plot and quantitatively by calculation of the Egger test (17).

### Subgroup analyses

We conducted subgroup analyses to test for interactions based on the duration of treatment (≤4 weeks, ≥6 weeks), type of AT (Scalp Acupuncture, body acupuncture), and stage of disease (Other Period,Subacute Period).

### Sensitivity analyses

We conducted sensitivity analyses to confirm the stability and credibility of the results of this meta-analysis using the Leave-One-Out method, which means omitting each study individually to exclude all factors that could have a significant impact on the pooled effect.

## Results

### Eligible studies and study characteristics

**Figure 1:**
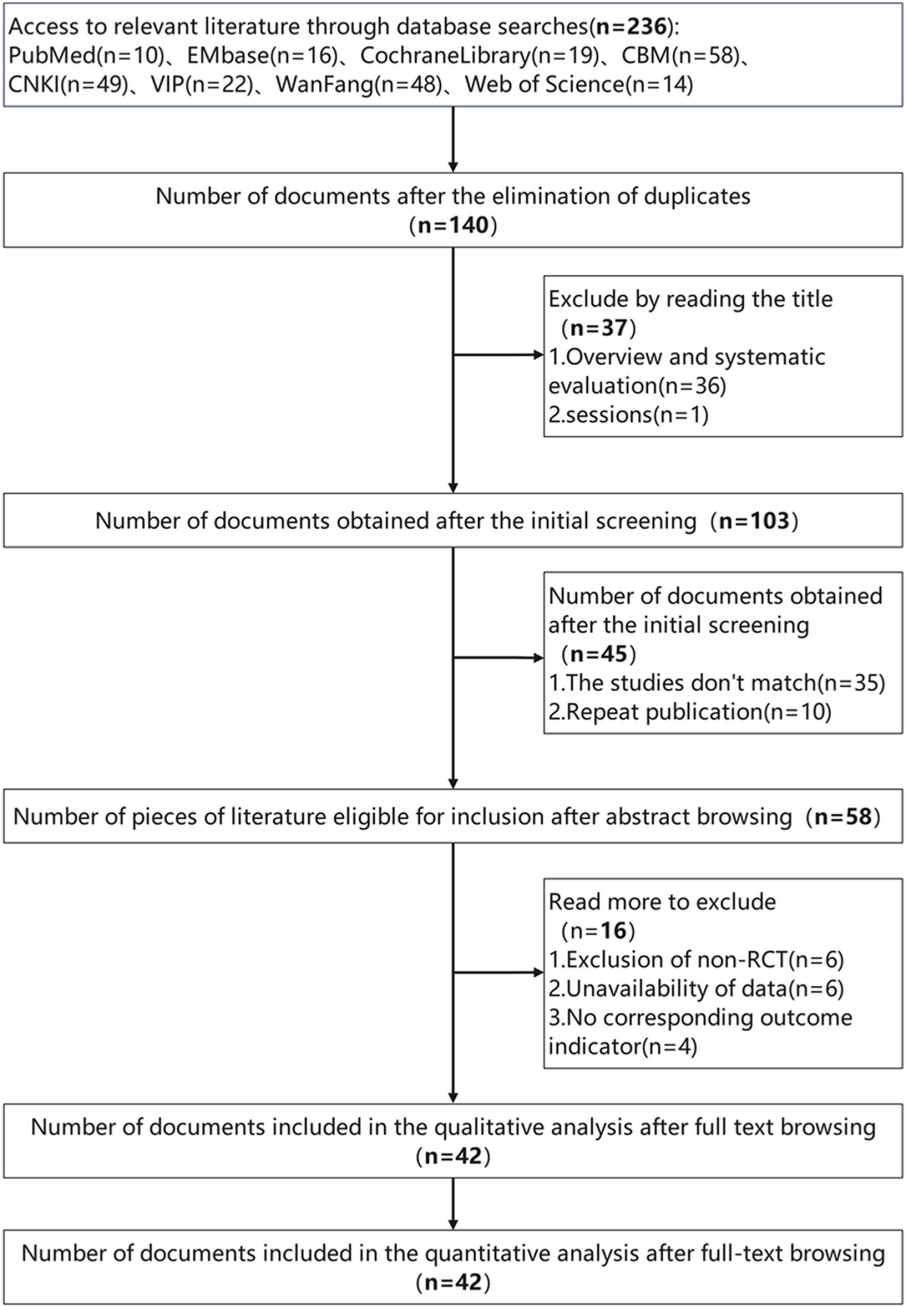
Flowchart of study selection for the meta-analysis.

After first screening 263 records, our final meta-analysis (shown in Figure 1) included 42 suitable trials (12, 18–58). Together, these investigations included 3,340 people ranging in age from 50 to 80 years. While the control groups received MT, AT, or rehabilitation therapy (RT) alone, with treatment durations ranging from a minimum of 2 weeks to a maximum of 12 weeks, the intervention groups received both MT and AT. Supplementary Spreadsheets 3, 4 contain further information on these studies; Supplementary eTables 1, 2 evaluate the risk of bias across the trials. Only one study was found to be highly biassed; the other studies showed low to moderate hazards, implying a largely consistent body of evidence.

### Primary outcome: FMA

**Figure 2:**
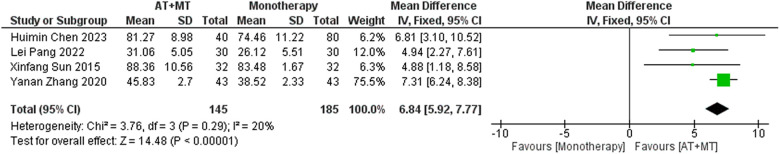
Forest plot of FMA-T outcomes comparing AT + MT with control interventions.

**Figure 3:**
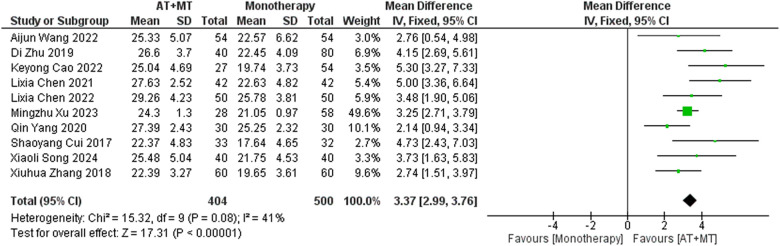
Forest plot of FMA-UE outcomes comparing AT + MT with control interventions.

**Figure 4:**
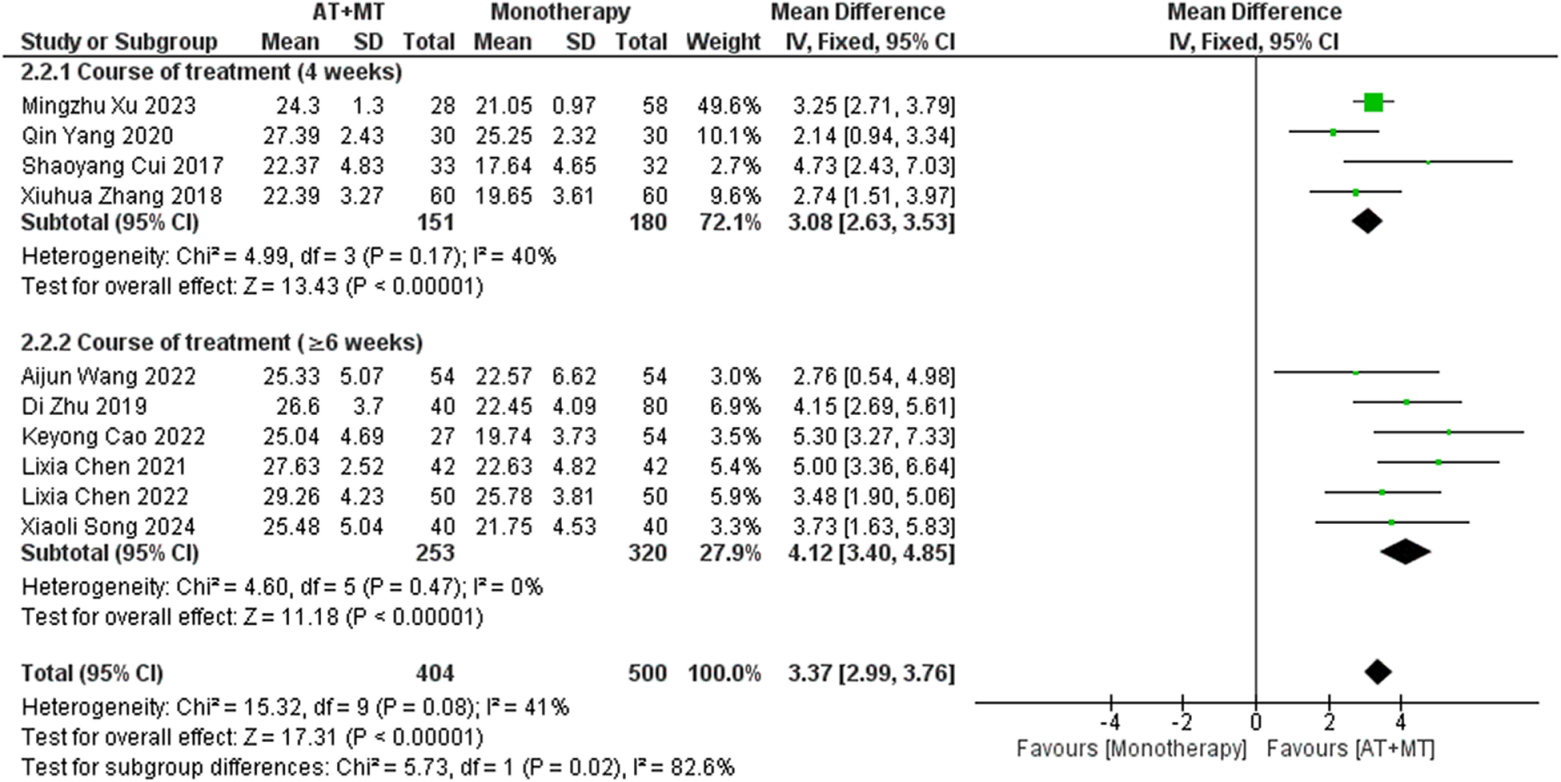
Forest plot of FMA-L outcomes comparing AT + MT with control interventions.

Data on FMA-T, FMA-UE, and FMA-L were reported in 4, 23, and 10 trials, respectively. FMA-T, FMA-UE, and FMA-L were higher in the intervention group than in the control group by 6.84 (95% CI 5.92–7.77, I^2^ = 20%; Figure 2), 6.77 (95% CI 5.60–7.93, I^2^= 87%; Figure 3), and 3.37 (95% CI 2.99–3.76, I^2^ = 41%; Figure 4), respectively. Among them, Egger's test of FMA-T (*P* = 0.1285) did not find the existence of publication bias (Supplementary eFigure S3). The Egger's test of FMA-UE and FMA-L (*P* < 0.05) found the existence of publication bias; then the cut-and-patch method was needed, and after the cut-and-patch, it was found that the combined effect sizes of FMA-UE and FMA-L were not significantly changed, implying that there was a slight publication bias. change, implying the existence of a slight publication bias, which does not affect the stability of the study results (Supplementary eFigures 4, 5). In sensitivity analyses, the results of meta-analyses of FMA-T, FMA-UE, and FMA-L were robust (Supplementary eFigures 6–S8).

**Figure 5:**
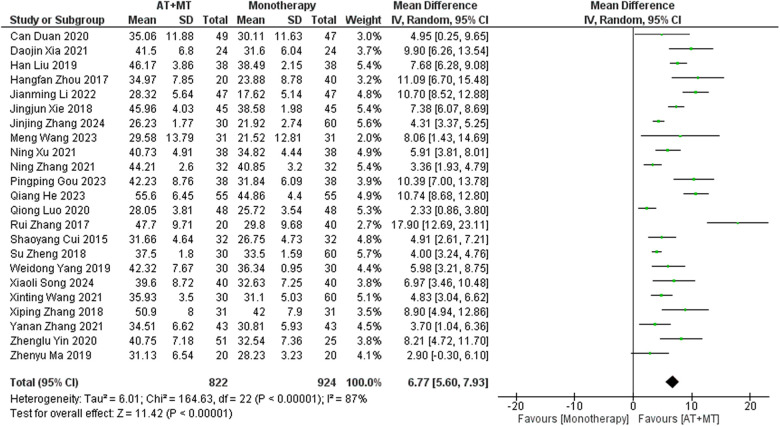
Subgroup analysis of treatment duration on FMA-L outcomes.

**Figure 6:**
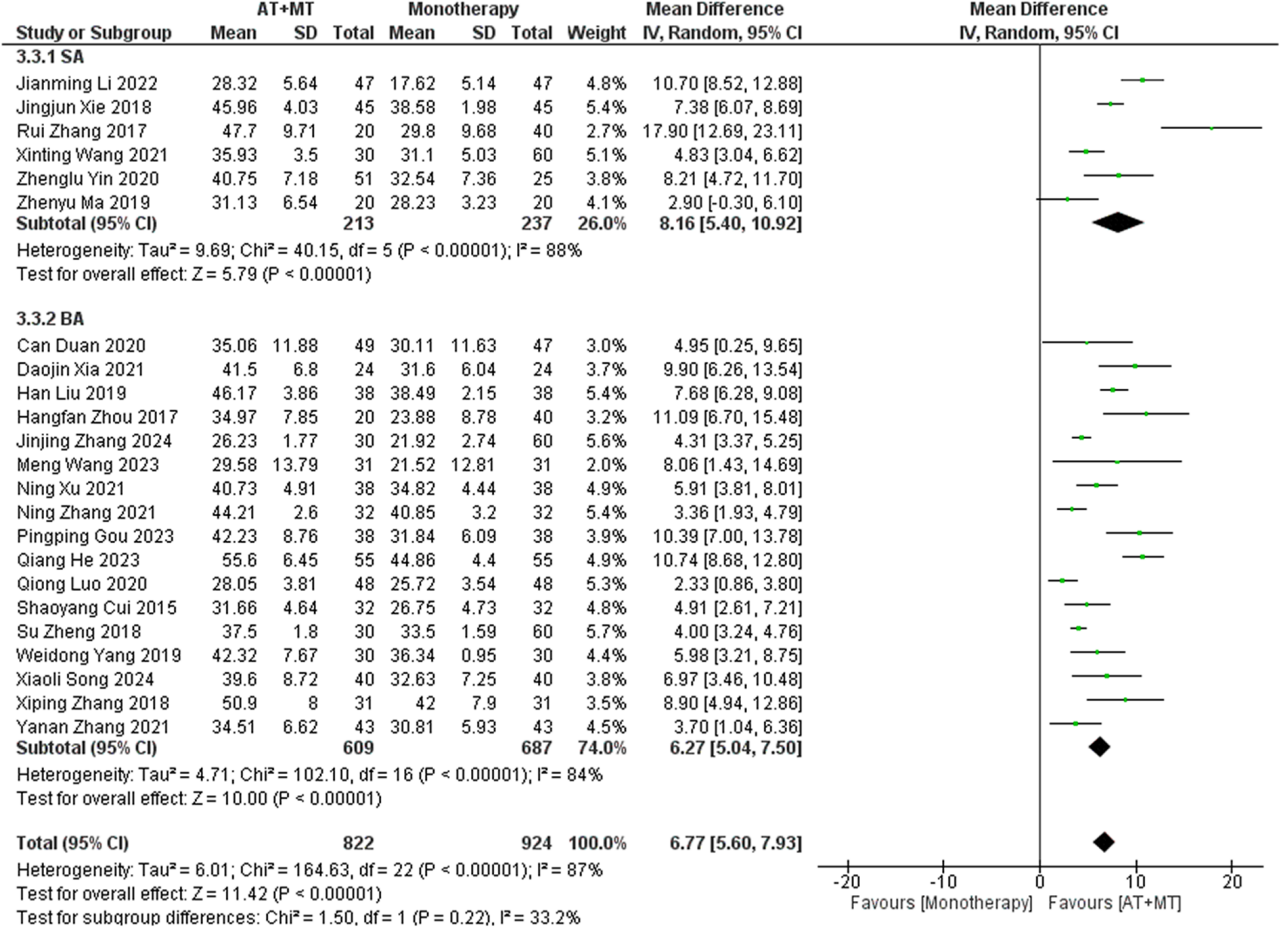
Subgroup analysis of treatment duration on FMA-UE outcomes.

**Figure 7:**
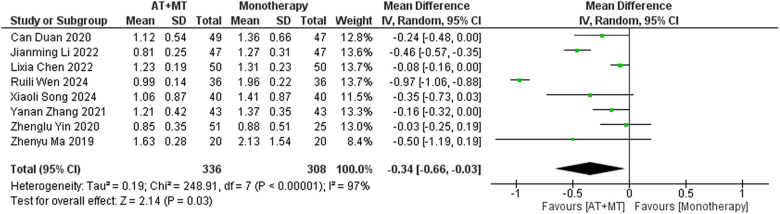
Subgroup analysis of AT type (SA vs. BA) on FMA-UE outcomes.

**Figure 8:**
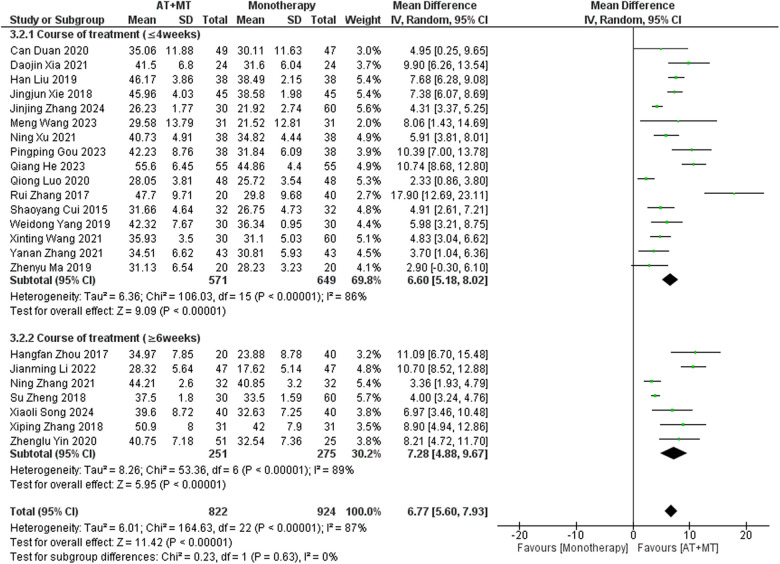
Subgroup analysis of AT type (SA vs. BA) on FMA-L outcomes.

**Figure 9:**
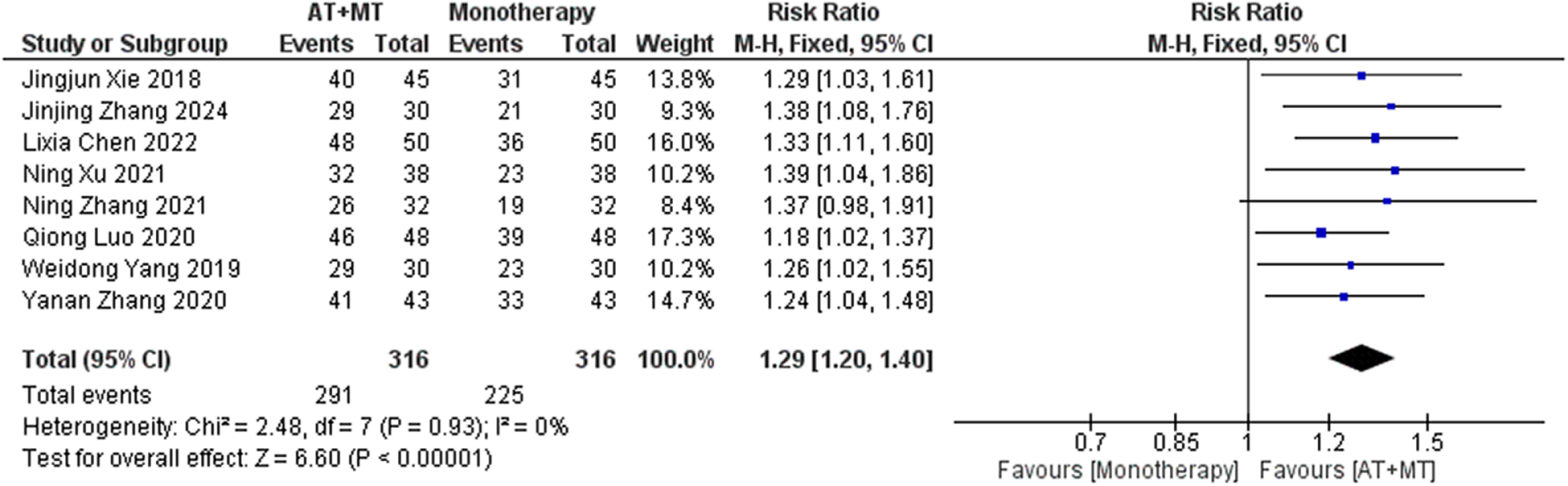
Subgroup analysis of disease stage (OP vs. SP) on FMA-L outcomes.

**Figure 10:**
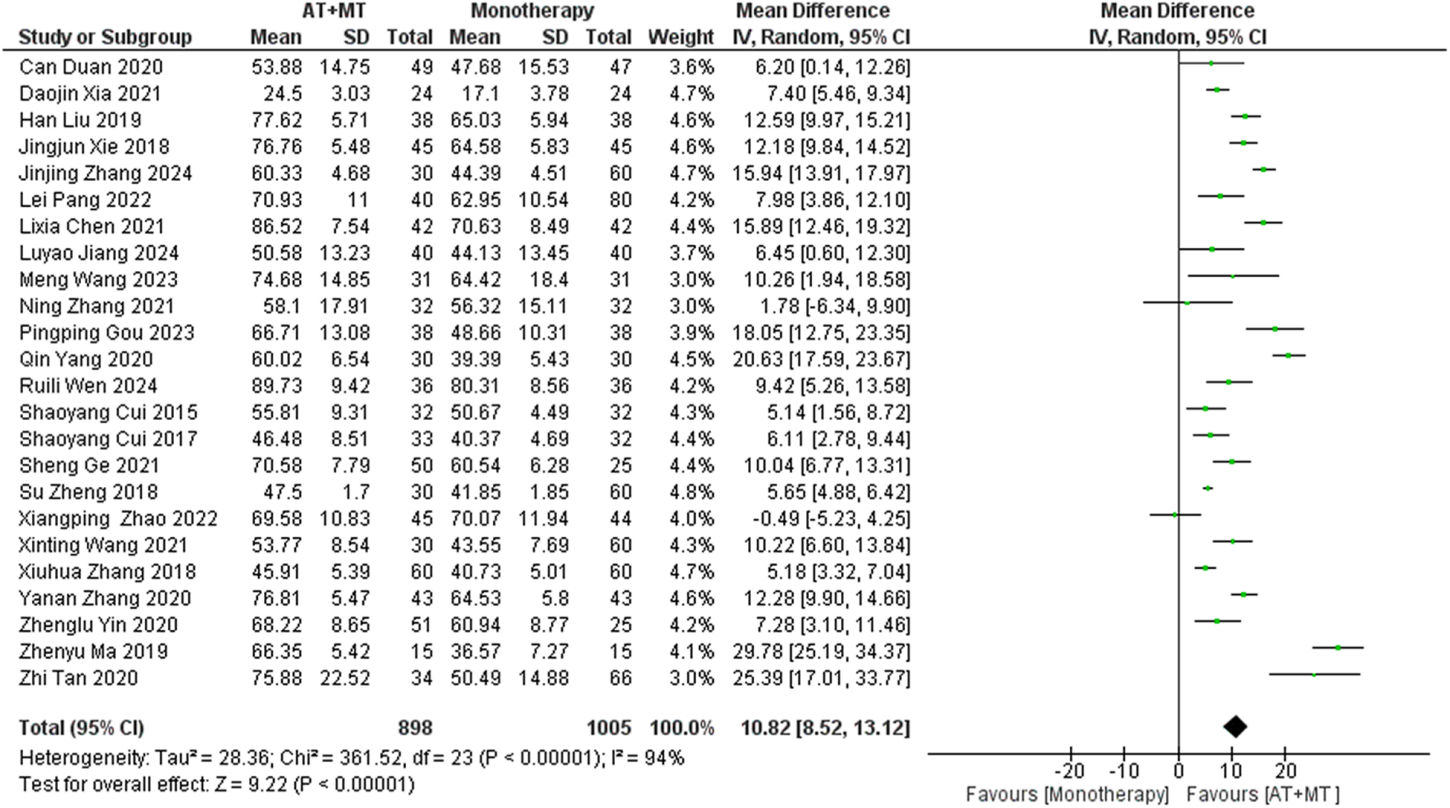
Subgroup analysis of disease stage (OP vs. SP) on FMA-UE outcomes.

Subgroup analyses found that the efficacy of FMA-L for ≥6 weeks of treatment was significantly higher than that for ≤4 weeks of treatment (interaction *P* = 0.02; Figure 5), and the efficacy was significant regardless of whether it was ≤4 weeks or ≥6 weeks; subgroup analyses found that there was no significant difference in the efficacy of FMA-UE for ≥6 weeks of treatment compared with that for ≤4 weeks of treatment (interaction *P* = 0.63; Figure 6), and the efficacy of FMA-UE for either ≤4 or ≥6 weeks was significant. was significant; subgroup analyses revealed no significant difference in FMA-UE and FMA-L efficacy between Scalp Acupuncture(SA) and Body Acupuncture(BA) (interaction P_FMA−UE_ = 0.22, P_FMA−L_ = 0.1; Figures 7, 8); subgroup analyses found no significant difference in FMA-L, FMA-UE between OP(Other Period) and SA (interaction P_FMA−L_ = 0.38, P_FMA−UE_ = 0.20, Figures 9, 10), which was significant regardless of OP or SP(Subacute Period).

### Secondary outcome: MBI, MAS, and total effective rate

**Figure 11:**
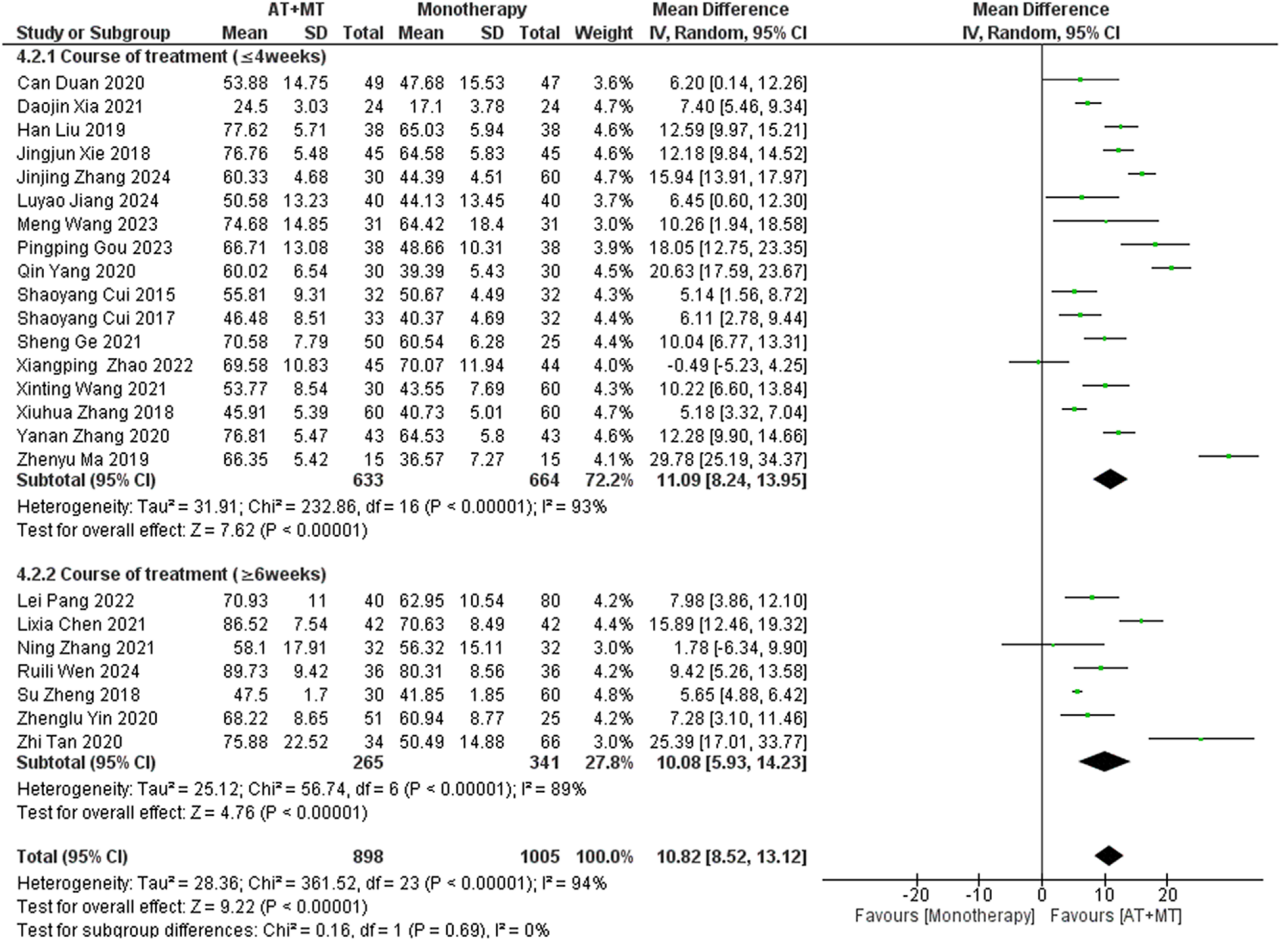
Forest plot of MBI outcomes comparing AT + MT with control interventions.

**Figure 12:**
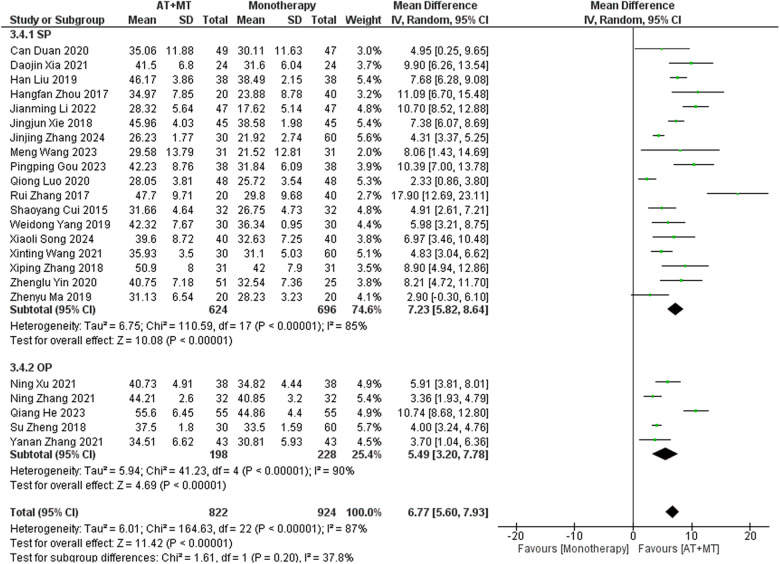
Forest plot of MAS outcomes comparing AT + MT with control interventions.

**Figure 13:**
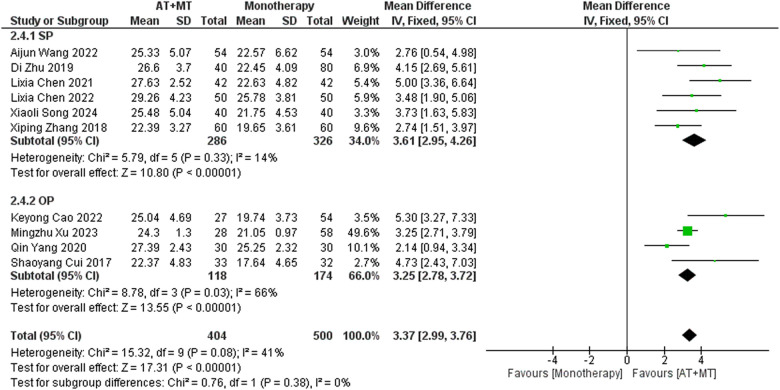
Forest plot of total effective rate comparing AT + MT with control interventions.

Data on MBI, MAS, and total effective rate were reported in 24, 8, and 8 trials, respectively. The MBI, MAS, and total effective rate were higher in the intervention group than in the control group by 10.82 (95% CI 8.52–13.12, I^2^ = 94%; Figure 11), −0.34 (95% CI −0.66–0.03, I^2^ = 97%; Figure 12), 1.29 (95% CI 1.20–1.40, I^2^ = 0%; Figure 13). Among them, the theorem's test for MBI, MAS, and total effective rate (P_MBI_ = 0.7759, P_MAS_ = 0.9443, and P_Total effective rate_ = 0.2203) did not reveal the presence of publication bias (Supplementary eFigures S9–S11). In the sensitivity analyses, the meta-analysis results of MBI, MAS, and total effective rate were robust (Supplementary Figures 12–S14).

**Figure 14:**
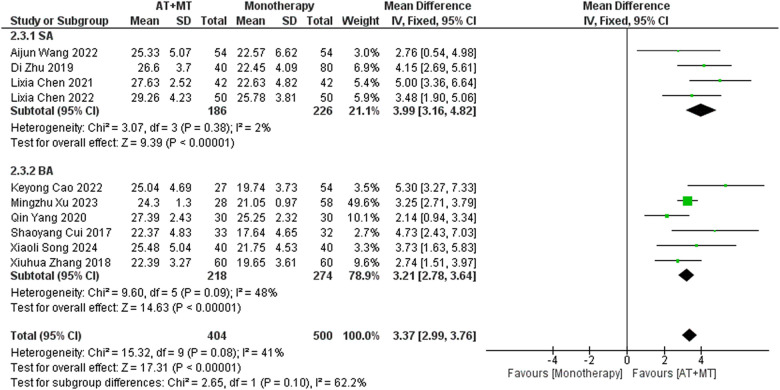
Subgroup analysis of treatment duration on MBI outcomes.

**Figure 15:**
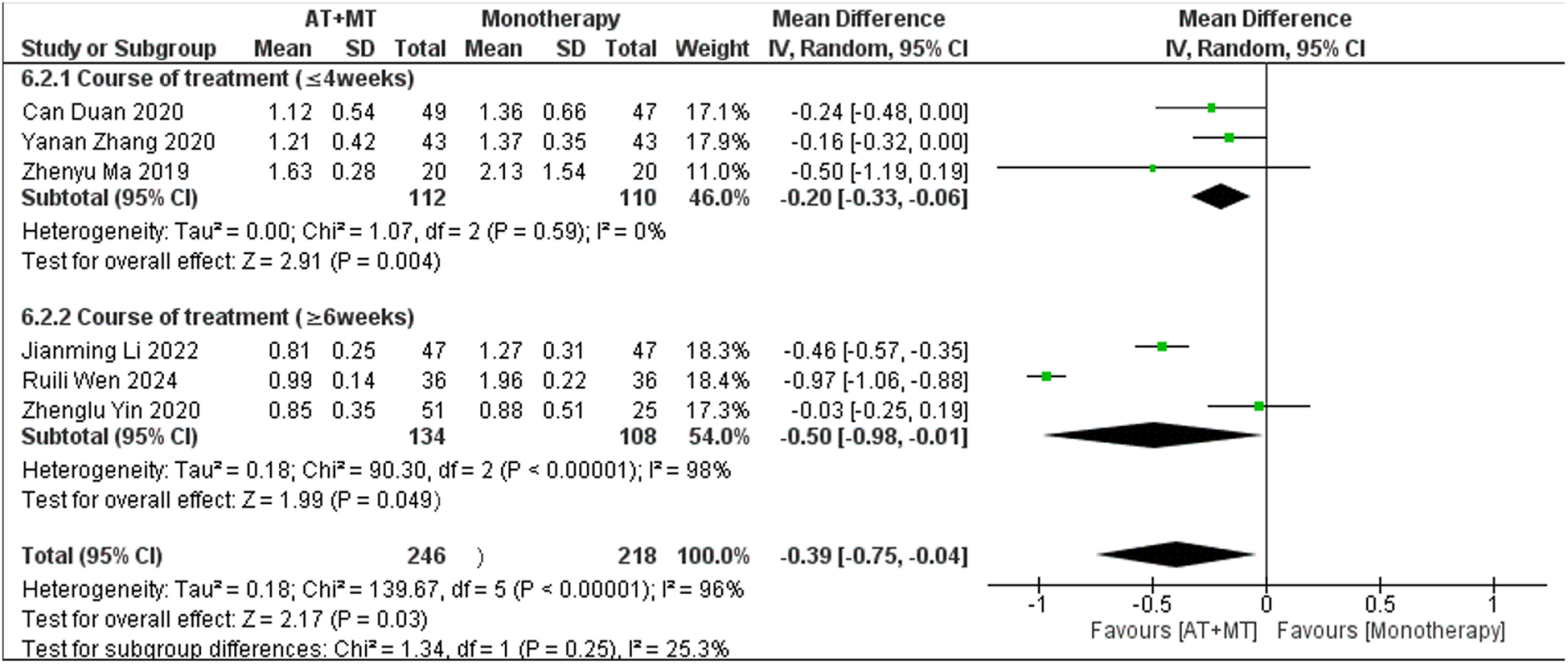
Subgroup analysis of treatment duration on MAS upper limb outcomes.

**Figure 16:**
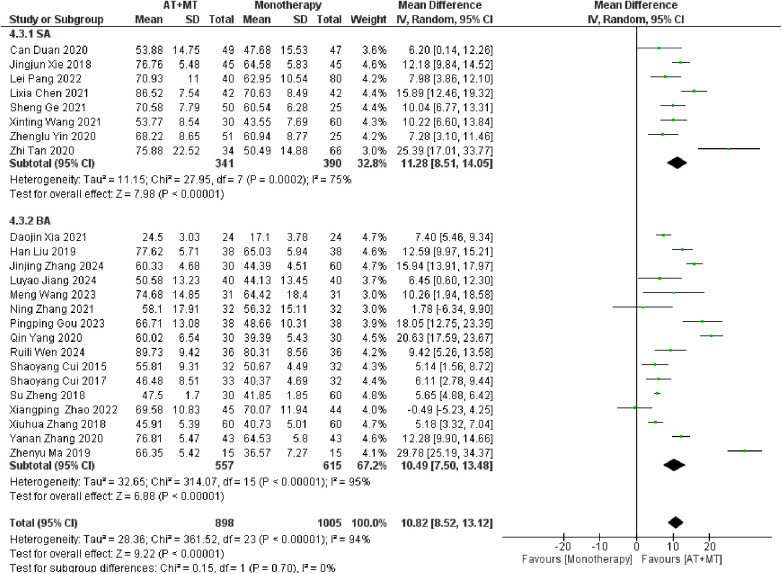
Subgroup analysis of AT type (SA vs. BA) on MBI outcomes.

**Figure 17:**
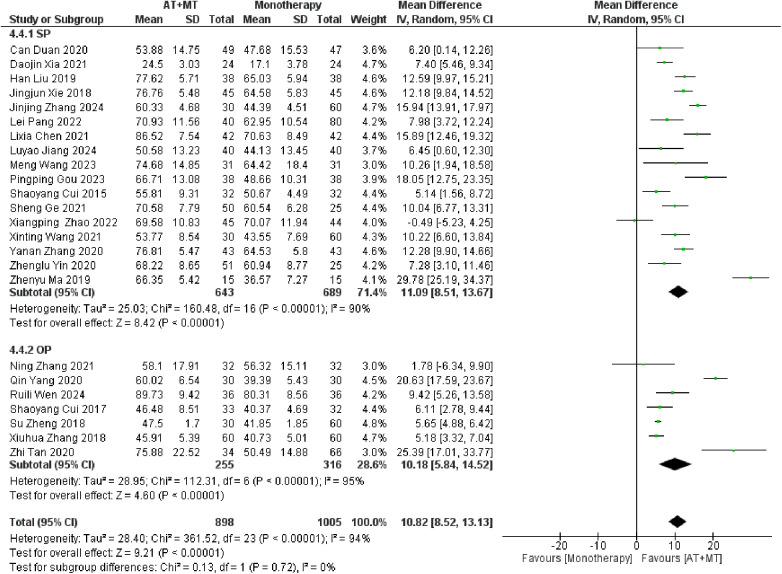
Subgroup analysis of disease stage (OP vs. SP) on MBI outcomes.

**Figure 18:**
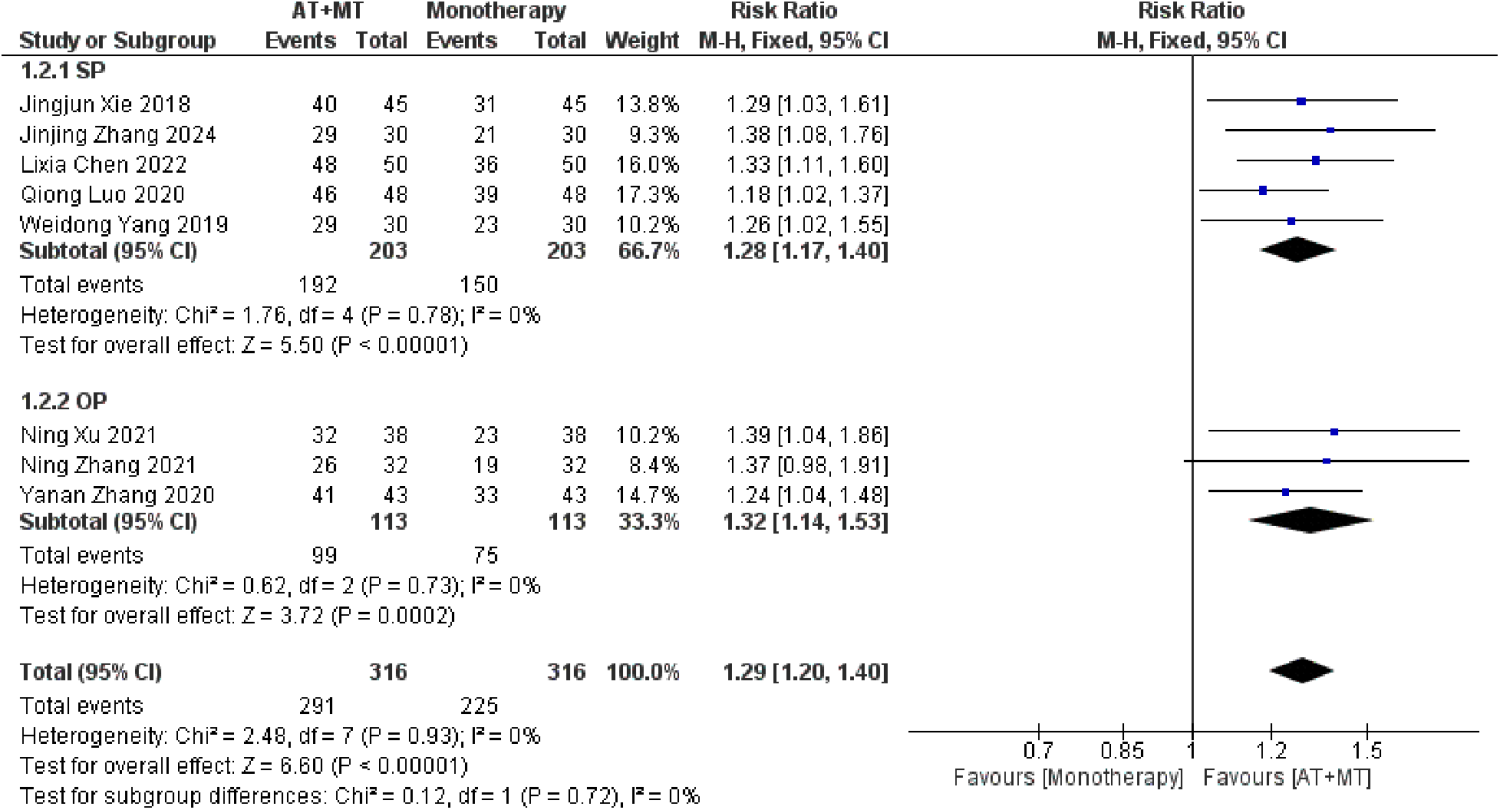
Subgroup analysis of disease stage (OP vs. SP) on Total effective rate outcomes.

There was no significant difference between the groups in how well MBI worked for MAS upper limb portion of ≥6 weeks vs. ≤4 weeks (interaction P_MBI_ = 0.69, P_MAS_ upper limb portion = 0.25; Figures 14, 15), and it was effective no matter what time period was used. There was also no significant difference between the groups in how well MBI worked for SA vs. BA (interaction *P* = 0.70; Figure 16), and it was effective no matter which group it was in. There was also no significant difference between the groups in how well it worked for OP and SP (P_MBI_ = 0.72, P_Total effective rate_ = 0.72, Figures 17, 18), and it was effective no matter which group it was in.

## Discussion

In this meta-analysis of 42 randomized controlled trials with a total of 3,440 participants, the results showed that AT combined with MT for the treatment of post-stroke limb dyskinesia resulted in a significantly higher Total effective rate compared to the control group (RR 1.29, 95% confidence interval 1.20–1.40); a significant improvement in FMA-T compared to control (MD 6.84, 95% confidence interval 5.92–7.77); a significant improvement in FMA-UE compared to control (MD 6.77, 95% confidence interval 5.60–7.93); FMA-L was significantly better than control (MD 3.37, 95% confidence interval 2.99–3.76); MBI was significantly improved (MD 10.82, 95% confidence interval 8.52–13.12), and MAS was significantly improved (MD −0.34, 95% confidence interval −0.66 t −0.03) compared with the control group.

### Principal findings and comparison with other studies

The findings of this study, which examined the use of AT paired with MT for treating motor deficits after a stroke, indicate that the overall results are not significantly different from those reported in a prior systematic review. Our study stands out methodologically as it incorporates nine randomized controlled studies (19, 21–23, 25, 35, 46, 47, 53) that were published after January 2023, representing 21.4% of the more recent trials. Moreover, these newer trials account for 19.7% of the total participants (676/3,440). This study further explored the relationship between treatment duration, types of AT, and disease staging with treatment outcomes through subgroup analysis.

Our study found that longer treatment durations significantly improved FMA-L outcomes in patients (interaction *P* = 0.02; Figure 5). This improvement may be due to the need for sustained training to achieve neural adaptations and muscle endurance in the lower limbs. Additionally, lower limb activities, such as walking and standing, involve more basic motor patterns and are relatively simpler in terms of motor control, making them more responsive to extended therapy. The longer therapy duration also provides time to gradually rebuild gait patterns and posture, which are important for functional recovery.

The results for FMA-UE, MBI, and MAS upper limb sections did not show significant correlations between treatment duration and treatment effects (interaction PFMA-UE = 0.63, PMBI = 0.70, PMAS upper limb = 0.25; Figures 6, 14, 15). This suggests that extending therapy may not be enough to improve upper limb motor function and MBI recovery. The modest effects on upper limb outcomes may be due to the complexity of upper limb rehabilitation, which involves finer motor control and neural signal modulation for the hands and arms (59–61). Furthermore, MBI reflects overall daily living abilities, which can be influenced by factors beyond motor function, such as cognitive status and environmental support. These factors may limit the extent to which extended therapy alone can lead to substantial improvements in MBI scores.

Our study found that there was no significant correlation between AT types and treatment effects for FMA-UE, FMA-L, and MBI (interaction P_FMA−UE_ = 0.22, P_FMA−L_ = 0.1, P_MBI_ = 0.70; Figures 7, 8, 16). SA emphasizes stimulation of specific scalp points, possibly involving indirect effects on brain functional areas through the nervous system (43, 62). In contrast, BA directly targets body points, potentially relying more on direct neural and physiological effects, such as muscle contraction regulation (63). Although theoretically these methods differ, our study did not demonstrate significant treatment efficacy differences among them. Despite the lack of observable differences in outcomes, this indirectly suggests that SA and BA may be equivalent in specific cases. Future studies should expand sample sizes and design more targeted trials to further explore differences in AT types for treating post-stroke limb functional impairments.

Our study results indicate that combined therapy for stroke patients did not significantly affect limb function recovery across different stages. Specifically, we did not observe significant differences in promoting lower limb function recovery between early intervention during the subacute phase and intervention during other phases. This finding contradicts previous research, which commonly indicates that early subacute phase intervention leads to improved recovery outcomes because of its benefits in brain plasticity and treatment time windows (60). Nevertheless, our findings indicate that while early intervention may have theoretical benefits, the actual rehabilitation procedures may be influenced by additional factors that impact the outcomes of recovery. The factors include personal variances, different approaches of treatment, and the degree of help provided during the rehabilitation process. The interaction of these elements could lead to the limited or negligible influence of the intervention timing on the recorded recovery results in our research. Moreover, our study emphasizes the need of paying more attention to the individualized rehabilitation demands and making judgments for stroke patients at several phases of clinical activity.

Many studies have shown that MT or AT alone can produce beneficial effects. For example, Madhoun et al. (64) reported that MT aids in upper limb motor recovery after stroke. Similarly, Yang et al. (65) found that acupuncture activates relevant neurons and enhances motor function. However, combining AT and MT has been shown to be more effective than either therapy alone for post-stroke movement disorders (25). Our systematic evaluation further confirmed the strong therapeutic potential of this combined approach, particularly in improving motor function. This provides a solid foundation for future studies with larger and more diverse populations.

### Strengths and limitations

The main limitation of this study is that the assessments were derived from subjective scales. In addition, the use of subjective scales in the assessment process may have contributed to some variability in results, as assessors might not have followed the uniform procedure strictly, even though standardized guidelines exist for scales like the FMA. The assessors were independent individuals with unique expertise and experience from a variety of trials.Secondly, subjective measures include various assessment factors but lack specificity and detail in capturing treatment effects (66). This limits their ability to provide a comprehensive evaluation. Furthermore, a single subjective scale cannot fully reflect individual differences and changes in patient conditions. Thus, it is necessary to integrate objective indicators, such as EMG systems, gait analysis, and fMRI, to gain a more comprehensive view. However, due to the high cost and limited accessibility of these devices, subjective scales remain the primary assessment method (67–70).

The other limitations of this study are as follows. Due to the different clinical presentations of stroke patients, the AT points or methods used during treatment vary greatly among individuals, making it difficult to avoid variations in treatment efficacy even within the same trial. Clinically, the subacute phase of stroke is considered one of the optimal times for treatment (60), leading researchers to focus more on the rehabilitation of patients in the subacute phase during trials. As a result, only a limited number of studies included patients in the acute and chronic phases in this research.

The strength of this study lies in its ability to capitalize on the value of the available data. The subgroup analyses consisted of three components: duration of treatment, type of AT, and stage of disease. The data obtained from these subgroup analyses provide clinicians with reliable recommendations for making treatment decisions.

### Implications

The FMA series scale scores are the most significant outcome of this analysis, demonstrating that in our study, stroke patients experienced significant improvements in both upper and lower limb functions following combined AT and MT treatment. Furthermore, in our course-of-treatment subgroup analysis, we found that the efficacy of treatment for lower limb dysfunction is related to the duration of therapy, suggesting that the effects of the combined treatment plan can accumulate for the lower limbs.Conversely, the effectiveness of treatment for upper limb dysfunction was not significantly correlated with treatment duration, suggesting that if the course of treatment extends beyond a certain threshold (e.g., 6–8 weeks), other more effective strategies could be implemented. This approach can optimize resource allocation and reduce patient costs. Additionally, our subgroup analysis of AT types showed no significant difference in patient outcomes between using SA or BA. Clinically, as different patients have varying tolerances to AT, physicians can now choose different treatment plans for limb dysfunction based on the patient's tolerance to AT.In the disease staging subgroup analysis, there was no significant difference in upper or lower limb dysfunction, indicating that this treatment regimen is appropriate for stroke patients at any clinical stage.

In terms of clinical efficacy, our study shows that after combined AT and MT treatment, the clinical efficacy rate of stroke patients was higher compared to the control group. In the disease staging subgroup analysis, there was no significant difference in clinical efficacy rates among stroke patients at different stages, suggesting that this approach is suitable for efficacy in all stages of stroke, making this protocol a viable treatment throughout the entire clinical phase of stroke patients.

MBI is a vital observational measure that is associated with patients’ ability to do their everyday activities. The results of our study indicate that stroke patients experienced notable enhancements in their ability to do daily tasks after receiving a combination of AT and MT. Subgroup analysis, considering the period of treatment, revealed no notable disparities in the degree of improvement across patients. This implies that after an adequate duration of treatment (e.g., 6–8 weeks), exploring more potent alternative methods may help further enhance patients’ daily functioning. Doing so can prevent unnecessary medical resource allocation and reduce treatment costs for patients. There were also no significant differences between SA and BA versions of AT, indicating that healthcare providers can select the treatment approach based on the patient's individual tolerance and preference, without compromising therapeutic outcomes. In addition, when subgroup analysis was conducted based on illness stage, there were no notable disparities in the enhancement of daily living skills among stroke patients at all stages. This indicates that the treatment plan is appropriate for all stages of stroke. Thus, this therapy regimen offers a viable choice that can be utilized throughout all stages of stroke patients’ clinical progression.

MAS is a crucial observational indicator related to the degree of spasticity in patients’ limbs. Our study indicates that following combined AT and MT therapy, stroke patients showed significant improvements in the degree of limb spasticity. Subgroup analysis based on AT type revealed no significant differences in the degree of limb spasticity among patients, suggesting that clinical practitioners can tailor treatment approaches for spasticity issues based on individual patient tolerance to AT.

### Suggestions for future studies

In future research, it is crucial for scientists to carefully consider the complexities of trial design, with a main emphasis on optimizing the use of assessment tools to evaluate the efficacy of therapies. Implementing this method has the capacity to enhance the objectivity of outcome measurements, hence enhancing the accuracy and dependability of the trials. In addition, researchers should contemplate enlarging the sample sizes to encompass a greater number of patients other stage. Implementing this strategy would result in more accurate trial conclusions and facilitate the investigation of the treatment regimen's suitability for different stages of stroke.

## Conclusions

In summary, researchers have found that combined AT and MT therapy is effective as a novel treatment approach for addressing post-stroke motor impairments. Future studies of larger scale and greater precision are necessary to yield more accurate conclusions.

## Data Availability

The data analyzed in this study is subject to the following licenses/restrictions: we can't disclose it because it involves the patient’s privacy. Requests to access these datasets should be directed to Weihao Ke, 804791727@qq.com.

## References

[1] Correction to: 2024 heart disease and stroke statistics: a report of US and global data from the American Heart Association. Circulation. (2024) 149(19):1010–5. 10.1161/CIR.0000000000001247. Erratum for: *Circulation*. (2024) 149(8):450–5. 10.1161/CIR.0000000000001247 38709844

[2] Thijs L Voets E Denissen S Mehrholz J Elsner B Lemmens R Trunk training following stroke. Stroke. (2023) 54(9):2000–5. 10.1161/STROKEAHA.123.043490 37639516

[3] Zhang Y Zhao W Wan C Wu X Huang J Wang X Exoskeleton rehabilitation robot training for balance and lower limb function in sub-acute stroke patients: a pilot, randomized controlled trial. J Neuroeng Rehabil. (2024) 21(1):98. 10.1186/s12984-024-01391-0 38851703 PMC11162020

[4] World Health Organization (WHO). The top 10 Ccauses of Ddeath. Geneva: World Health Organization (WHO) (2020). Available online at: https://www.who.int/news-room/fact-sheets/detail/the-top-10-causes-of-death (accessed March 10, 2020).

[5] Dobkin BH. Strategies for stroke rehabilitation. Lancet Neurol. (2004) 3(9):528–36. 10.1016/S1474-4422(04)00851-8 15324721 PMC4164204

[6] National Institute for Health and Care Excellence (NICE). Stroke Rrehabilitation in Aadults. NICE guideline NG236. London: National Institute for Health and Care Excellence (NICE) (2023). Available online at: NICE Guidelines.

[7] Powers WJ Rabinstein AA Ackerson T Adeoye OM Bambakidis NC Becker K 2018 Guidelines for the early management of patients with acute ischemic stroke: a guideline for healthcare professionals from the American Heart Association/American stroke association. Stroke. (2018) 49(3):e46–110. 10.1161/STR.0000000000000158 29367334

[8] World Health Organization. Acupuncture: Review and Aanalysis Rreports on Ccontrolled Cclinical Ttrials. Geneva: WHO (2002).

[9] Lv Q Xu G Pan Y Liu T Liu X Miao L Effect of acupuncture on neuroplasticity of stroke patients with motor dysfunction: a meta-analysis of fMRI studies. Neural Plast. (2021) 2021:8841720. 10.1155/2021/8841720 34188677 PMC8192216

[10] Bonafede M Dick A Noyes K Klein JD Brown T. The effect of acupuncture utilization on healthcare utilization. Med Care. (2008) 46(1):41–8. 10.1097/MLR.0b013e3181589b7d 18162854

[11] Lee MT Chen CC Lu HL Hsieh YW. Comparisons of three different modes of digital mirror therapy for post-stroke rehabilitation: preliminary results of randomized controlled trial. Digit Health. (2024) 10:20552076241260536. 10.1177/20552076241260536 38846366 PMC11155361

[12] Judi ZK Lixuan W Junlin Z Tongcai T. Efficacy observation of mirror therapy combined with scalp acupuncture in rehabilitation training for lower limb function in patients post-stroke. Chin J Rehabil Med. (2019) 34(5):533–8. 10.3969/j.issn.1001-1242.2019.05.007

[13] Peng Y Li N Du X Zhang G Huang S Ma J. Acupuncture combined with mirror therapy for post-stroke dyskinesia: a meta-analysis and systematic review. Medicine (Baltimore). (2024) 103(26):e38733. 10.1097/MD.0000000000038733 38941386 PMC11466092

[14] Page MJ McKenzie JE Bossuyt PM Boutron I Hoffmann TC Mulrow CD The PRISMA 2020 statement: an updated guideline for reporting systematic reviews. Br Med J. (2021) 372:n71. 10.1136/bmj.n71 33782057 PMC8005924

[15] Shinichi A. Cochrane handbook for systematic reviews of interventions. Online Kensaku. (2014) 35:154–5.

[16] Guyatt GH Oxman AD Vist GE Kunz R Falck-Ytter Y Schünemann HJ GRADE: an emerging consensus on rating quality of evidence and strength of recommendations. Br Med J. (2008) 336:924–6. 10.1136/bmj.39489.470347.AD 18436948 PMC2335261

[17] Higgins JP Thompson SG. Quantifying heterogeneity in a meta-analysis. Stat Med. (2002) 21:1539–58. 10.1002/sim.1186 12111919

[18] Weidong Y Yanjie M. Observation on the efficacy of electroacupuncture combined with mirror therapy in treating stage I shoulder-hand syndrome post-stroke. Shanghai J Acupun Moxibus. (2019) 38(5):492–6. 10.13460/j.issn.1005-0957.2019.05.0492

[19] Meng W Yulin Q Hong L Long Y Hao W Tao Y. Efficacy of “regulating yin and yang” acupuncture combined with mirror therapy in treating wrist-hand dysfunction post-stroke. Chin J Phys Med Rehabil. (2023) 45(11):982–5. 10.3969/j.issn.1008-8296.2018.04.010

[20] Ning X Weihua D Yun G Weifang W. Efficacy analysis of moxibustion combined with mirror therapy-based upper limb force feedback motor control training on upper limb function recovery in patients with acute ischemic stroke. J Hunan Univ Chin Med. (2021) 41(9):1361–4. 10.3969/j.issn.1674-070X.2021.09.009

[21] Ruili W Qibo C Qiang L Li C Hong G Wanqian L. Effects of fu zheng bu tu acupuncture combined with task-oriented mirror therapy on upper limb function in elderly patients with hemiparesis post-stroke. Hebei J Trad Chin Med. (2024) 46(3):460–3. 10.3969/j.issn.1002-2619.2024.03.024

[22] Luyao J Qimei L Jiangbiao H Lujing Y Saixuan C Meili Z. Effects of hegu needling method combined with mirror therapy on hand function in patients during the recovery phase of stroke. Zhejiang Clin Med. (2024) 26(1):42–4.

[23] Huimin C Ting M Tian Y Jin S Yongqing K Haokun L Rehabilitation efficacy of interactive scalp acupuncture combined with mirror therapy on upper limb dysfunction in patients with flaccid hemiplegia post-stroke. Shenzhen J Integr Trad Chin Western Med. (2023) 33(6):4–7. 10.16458/j.cnki.1007-0893.2023.06.002

[24] Zhenglu Y Zhaoxiang M Sheng G Minjie Z Linghui H. Clinical observation of interactive scalp acupuncture combined with task-oriented mirror therapy in treating upper limb dysfunction in ischemic stroke hemiplegia. Chin Acupunc Moxibus. (2020) 40(9):918–22. 10.13703/j.0255-2930.20190819-0001 32959583

[25] Mingzhu X Run L Huaneng W Yixiao W Lu L Bihan W Effect of Jin San needle combined with mirror therapy on lower limb dysfunction in patients with cerebral infarction based on surface electromyography analysis. Chin Gen Pract. (2023) 26(17):2162–8. 10.12114/j.issn.1007-9572.2023.0064

[26] Xiangping Z Xiaojun W Xing L Jing W Yun L Yanhong M Effects of left-right complementary acupuncture based on mirror neuron theory on unilateral neglect after stroke. Massage Rehab Med. (2022) 13(19):34–7.

[27] Shaoyang C Mingzhu X Shenghui Z Xinsheng L Chunzhi T Shuhui W Effects of jin san needle combined with mirror therapy on upper limb and hand dysfunction in hemiplegic patients. Chin J Phys Med Rehab. (2015) 37(7):550–1. 10.3760/cma.j.issn.0254-1424.2017.08.006

[28] Rui Z Meilan Z Ying Y Bangliang L Lianhua W. Effect of mirror therapy combined with tongdu Xingshen acupuncture on upper limb function recovery in patients after stroke. Chin J Phys Med Rehab. (2017) 39(8):588–93. 10.19787/j.issn.1008-1879.2022.19.007

[29] Xiping Z Chen P Jianping X Fanglun S. Effect of mirror therapy combined with acupoint embedding on upper limb motor function recovery in patients with hemiplegia after stroke. Chin Rural Med. (2018) 25(21):16–7. 10.3969/j.issn.1674-6449.2017.04.020

[30] Hangfan Z Hanxiao Y. Efficacy of mirror therapy combined with acupuncture on upper limb function in early hemiplegic patients. Health Res. (2017) 37(4):427–9. 10.19787/j.issn.1008-1879.2022.22.004

[31] Keyong C Wenya X Qixun W Peng Z. Observation on the efficacy of mirror therapy combined with acupuncture therapy on ankle dorsiflexion ability of the affected side in stroke patients. Massage Rehab Med. (2022) 13(22):13–6. 10.3969/j.issn.1008-8849.2024.05.016

[32] Hu J Lin YS Xu CQ Lyu HY Xu SF. Effect of mirror therapy combined with neuromuscular electrical stimulation on the functional recovery of lower limbs in stroke patients with hemiplegia. Chin J Gen Prac. (2022) 20(7):1131–4. 10.16766/j.cnki.issn.1674-4152.002540

[33] Xinfang S Guirong X Renyang Z. Clinical study of mirror movement therapy on motor function recovery in patients with cerebral infarction. Chin J Neuroimmunol Neurol. (2015) 22(2):146–7. 10.3969/j.issn.1006-9771.2021.11.012

[34] Su Z Jing X Li P. Effect of staged acupuncture combined with mirror therapy on upper limb motor function in patients with cerebral infarction and SPECT study. Acupunct Clin J. (2018) 34(2):24–8. 10.1155/2021/9487319

[35] Song XL Yang XM Ran DW Ding Y. Efficacy of twelve needles of hands and feet combined with mirror therapy in the treatment of hemiplegia after stroke and its effect on hemorheology, basic fibroblast growth factor, and homocysteine. Modern J Integ Trad Chin Western Med. (2024) 33(5):685–9. 10.1016/j.rehab.2019.10.005

[36] Xia DJ Xiong XS Lü YQ Fan R Peng T. Effects of bilateral upper limb transcutaneous acupoint electrical stimulation on upper limb function in patients with subacute stroke and hemiplegia. Chin J Rehab Theory Prac. (2021) 27(11):1318–22. 10.1002/adhm.202100646

[37] Zhang JJ Xiao HB Yang J Chen RQ Zhu ZJ Wang LY Clinical observation of the efficacy of acupuncture combined with mirror therapy in the treatment of upper limb motor dysfunction in stroke patients. J Anhui Univ Trad Chin Med. (2024) 43(2):57–61. 10.3969/j.issn.2095-7246.2024.02.014

[38] Chen LX Li CJ Yang AR. Effect of retained needling in scalp acupuncture combined with mirror therapy on lower limb dysfunction and activities of daily living in stroke patients. Modern J Integr Trad Chin Western Med. (2022) 31(7):929–32. 10.3969/j.issn.1008-8849.2022.07.012

[39] Li JM Chen WR Dang H Li CX Wu JM. Intervention of combined scalp acupuncture cluster needling and mirror therapy on upper limb muscle tone and event-related potentials in elderly patients with acute cerebral infarction and hemiplegia. Chin J Gerontol. (2022) 42(15):3641–5. 10.3969/j.issn.1005-9202.2022.15.006

[40] Wang XT Hu Y Zhang DN Zhou Z Zhu CT. Effect of combined scalp acupuncture and mirror therapy on upper limb motor function recovery in patients with cerebral infarction and hemiplegia. J Trad Chin Med. (2021) 27(12):93–6. 10.13862/j.cnki.cn43-1446/r.2021.12.019

[41] Wang AJ Jin Y Lin L Fu TF. Effect of scalp acupuncture combined with mirror therapy on lower limb motor function in stroke patients. Chin J Phys Med Rehabil. (2022) 44(2):531–3. 10.3760/cma.j.issn.0254-1424.2022.02.008

[42] Ge S Yin ZL Meng ZX Zhang XB Huang LH Zhang MJ. Effect of scalp acupuncture plus mirror therapy on lower limb motor function and walking ability in patients with ischemic stroke. Shanghai J Acu-mox. (2021) 40(6):647–9. 10.13460/j.issn.1005-0957.2021.06.0647

[43] Pang L Fu XX. Clinical observation of gait in patients with hemiplegia after stroke treated with combined scalp acupuncture and mirror therapy. Shandong Med J. (2022) 016:062. 10.3969/j.issn.1002-266X.2022.16.018

[44] Chen LX Li CJ Wang TT Yang AR. Effects of scalp acupuncture combined with mirror therapy on motor function and living ability in patients with post-stroke lower-limb dysfunction. Shanghai J Acu-mox. (2021) 40(3):279–81. 10.13460/j.issn.1005-0957.2021.03.0279

[45] He Q Fan L Zhu J Lian TT. Effects of warm acupuncture combined with mirror therapy on functional recovery, homocysteine, and brain-derived neurotrophic factor in elderly patients with hemiplegia after cerebral infarction. Hainan Med J. (2023) 34(7):929–31. 10.3969/j.issn.1003-6350.2023.07.004

[46] Gou PP Wei XL Pan HY. Observation of the efficacy of combined warm needling moxibustion and mirror therapy in the treatment of shoulder-hand syndrome after stroke in elderly patients. Hainan Med J. (2023) 34(11):1556–9. 10.3969/j.issn.1003-6350.2023.11.007

[47] Luo Q Lü J. Effect of combined warm needling moxibustion and mirror therapy on limb function and balance ability in patients with hemiplegia after stroke. Clin Med Res Prac. (2020) 5(29):3. 10.19347/j.cnki.2096-1413.202029051

[48] Zhang YN Zheng P Yan X Chen Y. Acupuncture at Baxie points combined with mirror therapy for upper limb and hand function in post-stroke hemiplegia: a clinical study. Chin Rehabil Med. (2021) 36(6):353–6. 10.3870/zgkf.2021.06.008

[49] Duan C Li ZL Xia WG Zheng CJ Zhang YP Li SC. Effect of acupuncture combined with mirror therapy on upper limb motor function recovery after stroke. Neural Inj Funct Reconstr. (2020) 15(3):155–8. 10.16780/j.cnki.sjssgncj.2020.03.009

[50] Zhang YN Yan X Gao WN Zheng P. Effect of acupuncture combined with mirror therapy on upper limb motor function in early-stage stroke patients. World Latest Med Inf. (2020) 20(82):186–7. 10.3969/j.issn.1671-3141.2020.82.103

[51] Xie JJ Li JX Sun Q Ma YJ. Therapeutic observation of acupuncture plus mirror therapy for upper-limb dysfunction of post-stroke hemiplegia. Shanghai J Acu-mox. (2018) 37(5):494–8. 10.13460/j.issn.1005-0957.2018.13.0052

[52] Zhenyu M Meifei X Xiaofeng Y. Clinical study on scalp acupuncture combined with mirror therapy for spastic paralysis of upper limbs due to stroke. J New Chinese Med. (2019) 51:182–5. 10.13457/j.cnki.jncm.2019.01.048

[53] Liu H. Efficacy of acupuncture combined with mirror therapy in the treatment of upper limb dysfunction in patients with hemiplegia after stroke. Chin Med Eng. (2019) 27(9):58–9.

[54] Zhang N Qie SY Zheng GH Gong WJ. Effect of acupuncture combined with mirror visual feedback training on upper limb function and daily living ability in patients with hemiplegia after stroke. Shandong J Trad Chin Med. (2021) 40(2):157–61. 10.16295/j.cnki.0257-358x.2021.02.009

[55] Zhang XH Liang JH Huang RM Zhu LY. Observation on the efficacy and nursing effect of combined acupuncture and mirror therapy on the recovery of lower limb function after stroke. Sichuan J Trad Chin Med. (2018) 36(3):208–10. 10.3969/j.issn.1000-3649.2018.03.016

[56] Yang Q Ni L. Rehabilitation efficacy of combined acupuncture and mirror feedback therapy on lower limb spasticity in stroke patients. World J Trad Chin Med. (2020) 15(19):2983–7. 10.3969/j.issn.1673-7202.2020.19.027

[57] Tan Z Li ZR Tan ZC Li XQ Yu BL. Clinical observation of scalp acupuncture combined with mirror therapy in the treatment of unilateral neglect after stroke. J Guangzhou Univ Trad Chin Med. (2020) 37(12):2359–64. 10.13359/j.cnki.gzxbtcm.2020.12.016

[58] Shao-yang C Ming-zhu X Shu-hui W Chun-zhi T Xin-sheng L Peng-dong J Effect of acupuncture plus mirror therapy on lower-limb dysfunction in hemiplegia after cerebral infarction. Shanghai J Acu-Mox. (2017) 36:9–13. 10.13460/j.issn.1005-0957.2017.01.0009

[59] Twitchell TE. The restoration of motor function following hemiplegia in man. Brain. (1951) 74(4):443–80. 10.1093/brain/74.4.443 14895765

[60] Krakauer JW. Motor learning: its relevance to stroke recovery and neurorehabilitation. Curr Opin Neurol. (2006) 19(1):84–90. 10.1097/01.wco.0000200544.29915.cc 16415682

[61] Kwakkel G Kollen BJ van der Grond J Prevo AJ. Probability of regaining dexterity in the flaccid upper limb: impact of severity of paresis and time since onset in acute stroke. Stroke. (2003) 34(9):2181–6. 10.1161/01.STR.0000087172.16305.CD 12907818

[62] Zhang D Zou W Zhang B Guo P. Scalp acupuncture for post-stroke spastic hemiparesis: a systematic review and meta-analysis. Medicine (Baltimore). (2024) 103(9):e37167. 10.1097/MD.0000000000037167 38428878 PMC10906645

[63] Zhang R Lao L Ren K Berman BM. Mechanisms of acupuncture-electroacupuncture on persistent pain. Anesthesiology. (2014) 120(2):482–503. 10.1097/ALN.0000000000000101 24322588 PMC3947586

[64] Madhoun HY Tan B Feng Y Zhou Y Zhou C Yu L. Task-based mirror therapy enhances the upper limb motor function in subacute stroke patients: a randomized control trial. Eur J Phys Rehabil Med. (2020) 56(3):265–71. 10.23736/S1973-9087.20.06070-0 32214062

[65] Yang Y Wang Z Hu Q Long X Ma G Cui S Xu M Tang C Yang C. The short-term effects of Jin’s three needles in conjunction with mirror therapy on brain function in patients with upper limb disability following an ischemic stroke were evaluated using ReHo analysis. Medicine (Baltimore). 2024 103(27):e38707. 10.1097/MD.0000000000038707 38968538 PMC11224885

[66] Bernhardt J Hayward KS Kwakkel G Ward NS Wolf SL Borschmann K Agreed definitions and a shared vision for new standards in stroke recovery research: the stroke recovery and rehabilitation roundtable taskforce. Int J Stroke. (2017) 12(5):444–50. 10.1177/1747493017711816 28697708

[67] Schwartz MH Rozumalski A. The gait deviation index: a new comprehensive index of gait pathology. Gait Posture. (2008) 28(3):351–7. 10.1016/j.gaitpost.2008.05.001 18565753

[68] Bohannon RW. Hand-grip dynamometry predicts future outcomes in aging adults. J Geriatr Phys Ther. (2008) 31(1):3–10. 10.1519/00139143-200831010-00002 18489802

[69] Ward NS Brown MM Thompson AJ Frackowiak RS. The influence of time after stroke on brain activations during a motor task. Ann Neurol. (2004) 55(6):829–34. 10.1002/ana.20099 15174016 PMC3713311

[70] Farina D Merletti R Enoka RM. The extraction of neural strategies from the surface EMG: an update. J Appl Physiol. (2014) 117(11):1215–30. 10.1152/japplphysiol.00162.2014 25277737 PMC4254845

