# Supplementary material for: Efficacy of acupuncture combined with mirror therapy in the treatment of post-stroke limb movement disorders: a systematic review and meta-analysis of randomised controlled trials
Source: Front Rehabil Sci. 2024 Nov 7;5:1464502. doi: 10.3389/fresc.2024.1464502 (PMC11580039; doi:10.3389/fresc.2024.1464502)
Supplement: Supplementary file 1 [file Datasheet1.docx]

**Supplemental e-material**

eTable 1: Definitions of outcomes

eTable 2: Search strategy

eTable 3: Characteristics of included trials.

eTable 4:  Acupuncture characteristics of the included studies..

eFigure 1: Risk of bias summary

eFigure 2: Risk of bias graph

eFigure 3: Funnel plot of FMA-T

eFigure 4: Funnel plot of FMA-L

eFigure 5: Funnel plot of FMA-L

eFigure 6: FMA-T sensitivity analysis

eFigure 7:FMA-UE sensitivity analysis

eFigure 8: FMA-L sensitivity analysis

eFigure 9: Funnel plot of MBI

eFigure 10: Funnel plot of MAS

eFigure 11: Funnel plot of Total effective rate

eFigure 12: MBI sensitivity analysis

eFigure 13: MAS sensitivity analysis

eFigure 14: Total effective rate sensitivity analysis

**eTable 1: Definitions of outcomes**

| **Outcomes** | **Definitions** |
| --- | --- |
| FMA-T | Fugl-Meyer MotOR Function Rating Scale, total scORe 226. |
| FMA-UE | Upper extremity pORtion of the Fugl-Meyer MotOR Function Rating Scale with a total scORe of 66. |
| FMA-L | Lower extremity pORtion of the Fugl-Meyer MotOR Function Rating Scale with a total scORe of 34. |
| MAS | Clinical tools to assess the degree of muscle spasticity，0 points fOR Level 0, 1 point fOR Level I, 1.5 points fOR Level I^+^, 2 points fOR Level II, 3 points fOR Level III, and 4 points fOR Level IV. |
| MBI | Modified Barthel Index, a tool used to assess an individual's ability to perfORm activities of daily living (ADL) out of 100. |
| Total effective rate | PropORtion of treatment measures that are effective, expressed as a percentage. |

**eTable 2: Search strategy**

| **PubMed** | | |
| --- | --- | --- |
| 1 | (((((((((((((((("Stroke"[Mesh]) OR (Stroke*[Title/Abstract])) OR (Cerebrovascular Apoplexy[Title/Abstract])) OR (Apoplexy*[Title/Abstract])) OR (Vascular Accident, Brain[Title/Abstract])) OR (Brain Vascular Accident*[Title/Abstract])) OR (Vascular Accidents, Brain[Title/Abstract])) OR (Cerebrovascular Stroke*[Title/Abstract])) OR (Stroke*, Cerebrovascular[Title/Abstract])) OR (CVA (Cerebrovascular Accident[Title/Abstract]))) OR (CVAs (Cerebrovascular Accident[Title/Abstract]))) OR (Acute Stroke*[Title/Abstract])) OR (Stroke*, Acute[Title/Abstract])) OR (Cerebrovascular Accident*[Title/Abstract])) OR (Acute Cerebrovascular*[Title/Abstract])) OR (Cerebral Stroke*[Title/Abstract])) OR (Apoplexy[Title/Abstract]) | 389,191 |
| 2 | ((((MirrOR Movement Therapies[Title/Abstract]) OR (Movement Therap*, MirrOR[Title/Abstract])) OR (Therap*, MirrOR Movement[Title/Abstract])) OR (MirrOR Therapy[Title/Abstract])) OR ("MirrOR Movement Therapy"[Mesh]) | 621 |
| 3 | (((((((((Acupuncture[MeSH]) OR (Acupuncture)) OR (Moxibustion)) OR (Needle)) OR (Electroacupuncture)) OR (Electro-acupuncture)) OR (Needle warming moxibustion)) OR (Scalp acupuncture)) OR (Abdominal acupuncture)) OR (Body acupuncture) | 224,234 |
| 4 | ((((((((Randomized controlled trial) OR (Controlled clinical trial)) OR (Randomized)) OR (Random allocation)) OR (Randomly)) OR (Placebo)) OR (Double-blind method)) OR (Single-blind method)) OR (Trials) | 2,931,452 |
| 5 | 1 and 2 and 3 and 4 and 5 | 10 |
| **Embase** | | |

| 1 | cerebrovascular accident'/exp | 461232 |
| --- | --- | --- |
| 2 | stroke:ab,ti | 516659 |
| 3 | stroke*:ab,ti | 531449 |
| 4 | cerebrovascular AND apoplexy:ab,ti | 1715 |
| 5 | apoplexy*:ab,ti | 4368 |
| 6 | vascular AND accident, AND brain:ab,ti | 11169 |
| 7 | brain AND vascular AND accident*:ab,ti | 2489 |
| 8 | vascular AND accidents, AND brain:ab,ti | 515 |
| 9 | cerebrovascular AND stroke*:ab,ti | 337811 |
| 10 | stroke*, AND cerebrovascular:ab,ti | 1 |
| 11 | 'cerebrovascular accident':ab,ti | 9094 |
| 12 | acute AND stroke*:ab,ti | 151886 |
| 13 | stroke*, AND acute:ab,ti | 1 |
| 14 | cerebrovascular AND accident*:ab,ti | 18048 |
| 15 | acute AND cerebrovascular*:ab,ti | 20736 |
| 16 | cerebral AND stroke*:ab,ti | 106753 |
| 17 | apoplexy:ab,ti | 4366 |
| 18 | 1 OR 2 OR 3 OR 4 OR 5 OR 6 OR 7 OR 8 OR 9 OR 10 OR 11 OR 12 OR 13 OR 14 OR 15 OR 16 OR 17 | 672930 |
| 19 | 'mirrOR therapy'/exp | 561 |
| 20 | mirrOR AND movement AND therapy:ab,ti | 504 |
| 21 | mirrOR AND movement AND therapies:ab,ti | 75 |
| 22 | movement AND therap*, AND mirrOR:ab,ti | 0 |
| 23 | therap*, AND mirrOR AND movement:ab,ti | 0 |
| 24 | mirrOR AND therapy:ab,ti | 3214 |
| 25 | 19 OR 20 OR 21 OR 22 OR 23 OR 24 | 3354 |
| 26 | 'acupuncture'/exp | 60365 |
| 27 | acupuncture:ab,ti | 40213 |
| 28 | moxibustion:ab,ti | 4931 |
| 29 | needle:ab,ti | 184050 |
| 30 | electroacupuncture:ab,ti | 8807 |
| 31 | 'electro acupuncture':ab,ti | 1426 |
| 32 | needle AND warming AND moxibustion:ab,ti | 90 |
| 33 | scalp AND acupuncture:ab,ti | 703 |
| 34 | abdominal AND acupuncture:ab,ti | 1190 |
| 35 | body AND acupuncture:ab,ti | 4870 |
| 36 | 26 OR 27 OR 28 OR 29 OR 30 OR 31 OR 32 OR 33 OR 34 OR 35 | 245052 |
| 37 | randomized AND controlled AND trial | 1195158 |
| 38 | controlled AND clinical AND trial | 1531382 |
| 39 | randomized | 1496802 |
| 40 | random AND allocation | 7102 |
| 41 | randomly | 580702 |
| 42 | placebo | 550157 |
| 43 | 'double blind' AND method | 21931 |
| 44 | 'single blind' AND method | 7636 |
| 45 | trials | 1123701 |
| 46 | 37 OR 38 OR 39 OR 40 OR 41 OR 42 OR 43 OR 44 OR 45 | 3134163 |
| 47 | 18 AND 25 AND 36 AND 46 | 16 |
| **Cochrane database** | | |
| 1 | MeSH descriptOR: [Acupuncture] explode all trees | 224 |
| 2 | Moxibustion | 6753 |
| 3 | Needle | 19851 |
| 4 | Electroacupuncture | 3815 |
| 5 | Electro-acupuncture | 798 |
| 6 | Needle warming moxibustion | 87 |
| 7 | Scalp acupuncture | 546 |
| 8 | Abdominal acupuncture | 942 |
| 9 | Body acupuncture | 2228 |
| 10 | 1 OR 2 OR 3 OR 4 OR 5 OR 6 OR 7 OR 8 OR 9 | 30475 |
| 11 | randomized controlled trial | 1106459 |
| 12 | controlled clinical trial | 1110644 |
| 13 | randomized | 1284525 |
| 14 | random allocation | 43102 |
| 15 | randomly | 338918 |
| 16 | placebo | 401314 |
| 17 | 'double blind' method | 193540 |
| 18 | 'single blind' method | 62969 |
| 19 | trials | 2183822 |
| 20 | 11 OR 12 OR 13 OR 14 OR 15 OR 16 OR 17 OR 18 OR 19 | 2184372 |
| 21 | Stroke* | 89455 |
| 22 | Cerebrovascular Apoplexy | 173 |
| 23 | Apoplexy* | 551 |
| 24 | Vascular Accident, Brain | 790 |
| 25 | Brain Vascular Accident* | 849 |
| 26 | Vascular Accidents, Brain | 78 |
| 27 | Cerebrovascular Stroke* | 22970 |
| 28 | Stroke*, Cerebrovascular | 0 |
| 29 | Cerebrovascular Accident | 18687 |
| 30 | Acute Stroke* | 22758 |
| 31 | Stroke*, Acute | 0 |
| 32 | Cerebrovascular Accident* | 18970 |
| 33 | Acute Cerebrovascular* | 8180 |
| 34 | Cerebral Stroke* | 12436 |
| 35 | Apoplexy | 551 |
| 36 | 21 OR 22 OR 23 OR 24 OR 25 OR 26 OR 27 OR 28 OR 29 OR 30 OR 31 OR 32 OR 33 OR 34 OR 35 | 93200 |
| 37 | MeSH descriptOR: [Stroke] explode all trees | 17550 |
| 38 | 36 OR 37 | 93564 |
| 39 | MeSH descriptOR: [MirrOR Movement Therapy] explode all trees | 35 |
| 40 | Movement Therap*, MirrOR | 0 |
| 41 | Therap*, MirrOR Movement | 0 |
| 42 | MirrOR Therapy | 1429 |
| 43 | 39 OR 40 OR 41 OR 42 | 1429 |
| 44 | 43 AND 38 AND 20 AND 10 | 19 |
| **Web of Since** | | |
| 1 | TS＝（MirrOR Movement Therapies OR Movement Therap*, MirrOR OR Therap*, MirrOR Movement OR MirrOR Therapy OR MirrOR Movement Therapy） | 15,246 |
| 2 | TS＝（Acupuncture OR Acupuncture OR Moxibustion OR Needle OR Electroacupuncture OR Electro-acupuncture OR Needle warming moxibustion OR Scalp acupuncture OR Abdominal acupuncture OR Body acupuncture） | [339,067](http://webofscience-clarivate-cn-s.swebvpn.cqmu.edu.cn:8118/wos/alldb/summary/5a78efbc-1464-4686-b4c3-6b4e58fc7dd6-f8be9f12/relevance/1) |
| 3 | TS＝（Stroke OR Stroke* OR Cerebrovascular Apoplexy OR Apoplexy* OR Vascular Accident, Brain OR Brain Vascular Accident* OR Vascular Accidents, Brain OR Cerebrovascular Stroke* OR Stroke*, Cerebrovascular OR CVA (Cerebrovascular Accident) OR CVAs (Cerebrovascular Accident) OR Acute Stroke* OR Stroke*, Acute OR Cerebrovascular Accident* OR Acute Cerebrovascular* OR Cerebral Stroke* OR Apoplexy） | 664,454 |
| 4 | ((((((((TS=(trials)) OR TS=('single blind' method)) OR TS=('double blind' method)) OR TS=(placebo)) OR TS=(randomly)) OR TS=(random allocation)) OR TS=(randomized)) OR TS=(controlled clinical trial)) OR TS=(randomized controlled trial) | 3,609,508 |
| 5 | 1 AND 2 AND 3 AND 4 AND 5 | 14 |
| **CBM database** | | |
| 1 | (Transient ischaemic attack) OR (Cerebral embolism) OR (Cerebral thrombosis) OR (Cerebral infarction) OR (Cerebral infarction) OR (Subarachnoid haemORrhage) OR (Cerebral stasis) OR (Cerebral haemORrhage) OR (Stroke) OR (Cerebral vascular accident) OR (Stroke) OR ("Stroke" [unweighted:Extended]) | 953556 |
| 2 | Acupuncture OR Body Acupuncture OR Acupuncture OR Head Acupuncture OR Warm Acupuncture OR Electroacupuncture | 344758 |
| 3 | (MirrOR Movement Therapy) OR (MirrOR Therapy) | 376 |
| 4 | 1 AND 2 AND 3 | 58 |

**eTable 3: Characteristics of included trials.**

| **Trial** | **Intervention** | **Control** | **Primary outcome** | **Sample size** | **Age** | **Clinical phase** |
| --- | --- | --- | --- | --- | --- | --- |
| Weidong Yang2019 | AT+MT | RT | TER,FMA-UE | 60 | E=51-72  C=54-75 | SP |
| Meng Wang2023 | AT+MT | AT | FMA-UE,MBI | 62 | E=57.58±10.38  C=59.90±13.26 | SP |
| Zhenglu Yin2020 | AT+MT | MT | FMA-UE,MAS,MBI | 76 | E1=55±10  E2=53±13  C=52±11 | SP |
| Ning Xu2021 | AT+MT | RT | FMA-UE,TER | 76 | E=61.31±7.51  C=61.44 ±7.74 | AP |
| Ruili Wen2024 | AT+MT | MT | MAS，MBI | 72 | E=65.47 ± 7.47  C=65.39 ± 7.43 | CP |
| Luyao Jiang2024 | AT+MT | AT | MBI | 40 | E=61.50±11.68  C=59.05±13.01 | SP |
| Minhui Chen2023 | AT+MT | MT | FMA-T | 60 | E=58.29 ± 2.31  C=58.36 ± 2.25 | SP |
| Mingzhu Xu2023 | AT+MT | C1=AT  C2=MT | FMA-L | 86 | E=59.3±10.6  C1=61.6±9.3  C2=59.1±9.3 | EP |
| Shaoyang Cui2015 | AT+MT | MT | FMA-UE、MBI | 64 | E=51.19±7.89  C=52.28±7.39 | SP |
| Rui Zhang2017 | AT+MT | C1=AT  C2=MT | FMA-UE | 60 | E=55.2±10.9  C1=54.9±11.3  C2=54.8±10.1 | SP |
| Xiping Zhang2018 | AT+MT | RT | FMA-UE | 62 | E=54.2±8.9  C=52.1±8.0 | SP |
| Hangfan Zhou2017 | AT+MT | C1=AT  C2=MT | FMA-UE | 60 | E=57.22±6.15  C1=54.20±5.03  C2=53.55±10.50 | SP |
| Keyong Cao2022 | AT+MT | C1=AT  C2=MT | FMA-L | 81 | E=64.07±10.25  C1=63.51±10.40  C2=62.37±11.49 | EP |
| Jun Hu2022 | AT+MT | AT | FMA-L | 64 | E=52.45±5.15  C=51.89±4.92 | SP |
| Di Zhu2019 | AT+MT | C1=AT  C2=MT | FMA-L | 120 | E=49±3.7  C1=54±1.9  C2=52±2.3 | SP |
| Xinfang Sun2015 | AT+MT | AT | FMA-T | 64 | E=61.2±10.4  C=61.4±10.5 | AP |
| Su Zheng2018 | AT+MT | C1=AT  C2=MT | FMA-UE,MBI | 90 | E=48.6 ± 4.3  C1=49.7±3.6  C2=50.3 ± 2.5 | CP |
| Xiaoli Song2024 | AT+MT | MT | MAS,FMA-L,FMA-UE | 80 | E=52.4±6.5  C=53.6±7.0 | SP |
| Daojin Xia2021 | AT+MT | MT | FMA-UE,MBI | 48 | E=56.20±9.91  C=56.50±7.90 | SP |
| Zhi Tan2020 | AT+MT | C1=AT  C2=MT | MBI | 100 | E=63.57±6.43  C1=62.27±6.18  C2=62.15±6.20 | AP |
| Lixia Chen2022 | AT+MT | MT | FMA-L,MAS,TER | 100 | E=64.2±8.8  C=63.8±7.8 | SP |

| Jianming Li2022 | AT+MT | RT | MAS，FMA-UE | 94 | E=68.35±4.16  C=67.56±3.77 | SP |
| --- | --- | --- | --- | --- | --- | --- |
| Zhenyu Ma 2019 | AT+MT | MT | FMA-UE、MAS、MBI | 40 | E=67.2±5.7  C=65.4±4.8 | SP |
| Xiangping Zhao2022 | AT+MT | AT | MBI | 89 | E=55.79±5.85  C=56.25±4.79 | SP |
| Xinting Wang2021 | AT+MT | C1=RT  C2=MT | FMA-UE,MBI,TER | 90 | E=53.97±8.89  C1=54.80±5.90  C2=56.60±9.44 | SP |
| Aijun Wang2022 | AT+MT | RT | FMA-L | 108 | E=62.20±9.17  C=63.75±8.56 | SP |
| Sheng Ge2021 | AT+MT | MT | MBI | 75 | E=54±12  C1=55±11  C2=55±11 | SP |
| Lei Pang2022 | AT+MT | C1=AT  C2=MT | MBI,FMA-T | 120 | E=62. 71±9.60  C1=62.68±9.64  C2=62. 64± 9. 58 | SP |
| Lixia Chen2021 | AT+MT | MT | MBI,FMA-L | 84 | E=56±12  C=58±11 | SP |
| Qiang He2023 | AT+MT | MT | FMA-UE | 110 | E=66.83±3.16  C=66.13±3.52 | AP |
| Pingping Gou2023 | AT+MT | RT | MBI,FMA-UE,TER | 76 | E=74.04±6.26  C=73.87±6.14 | SP |
| Qiong Luo2020 | AT+MT | MT | FMA-UE,TER | 96 | E=63.28±3.23  C=63.04±3.16 | SP |
| Yanan Zhang2021 | AT+MT | MT | FMA-UE,MAS | 86 | E=59.88±6.63  C=58.51±6.16 | EP |
| Can Duan2020 | AT+MT | MT | FMA-UE,MAS,MBI | 96 | E=58±10  C=60±11 | SP |
| Yanan Zhang2020 | AT  +MT | MT | FMA-T,MBI,ETR | 86 | E=51.93±6.57  C=52.07±6.62 | SP |
| Jinjing Zhang2024 | AT+MT | C1=AT  C2=MT | FMA-UE,MBI,ETR | 90 | E= 58.57 ±12.25  C1=58.96±12.00  C2=58.37±10.97 | SP |
| Han Liu2019 | AT+MT | MT | FMA-UE,MBI | 76 | E=59.82±3.41  C=59.71±3.68 | EP |
| Ning Zhang2021 | AT+MT | AT | FMA-UE,MBI,ETR | 64 | E=58.81±10.94  C=56.37±9.53 | AP |
| Shaoyang Cui2017 | AT+MT | MT | FMA-L,MBI | 65 | E=51±4  C=53±4 | AP |
| Xiuhua Zhang 2018 | AT+MT | AT | FMA-L,MBI | 120 | E=61.79±2.07  C=61.85±2.35 | EP |
| Qin Yang 2020 | AT+MT | MT | FMA-L,MBI | 60 | E=60 ± 6.98  C=59 ± 7.23 | CP |

| Jingjun Xie 2018 | AT+MT | MT | FMA-UE,MBI,TER | 90 | E=56±8  C=54±6 | SP |
| --- | --- | --- | --- | --- | --- | --- |

AT = acupuncture therapy; MT = mirror therapy; RT = rehabilitation therapy; E=Experimental Group(E1=Experimental Group1,E2=Experimental Group2);C=Control Group(C1=Control Group1,C2=Control Group2);TER=Total effective rate;AP=acute phase;SP=subacute phase;EP=Early chronic phase;CP=chronic phase.

**eTable 4:  Acupuncture characteristics of the included studies.**

| Trial | Type of acupuncture | Acupoint formula | Stimulation response | Dosage | Treatment duration |
| --- | --- | --- | --- | --- | --- |
| Weidong Yang2019 | BA+ES | LI11,SJ5,LI4,LI5,LI15,TE15,LU15 | 1-4mA,2/15Hz | 5 times a wk,30 min each time | 4 weeks |
| Meng Wang2023 | BA | LI15,LI14,Ex-UE12,HT2,LI11,LI10,SJ5,LI4,LU10,SI3 | Deqi sensation, twirling | 6 times a wk,30 min each time | 2 weeks |
| Zhenglu Yin2020 | SA | Top 1/5, middle 2/5 of the anterior parietal temporal slope and top 1/5, middle 2/5 of the posterior parietal temporal slope | Twirling | 5 times a wk,40 min each time | 8 weeks |
| Ning Xu2021 | BA | GV14-GV2 | Moxibustion | 6 times a wk,15 min each time | 2 weeks |
| Ruili Wen2024 | BA | LI11,ST36,SP6,LI4,BL40,LI15，CV6,CV4,BL23,BL20,BL18 | Deqi sensation, twirling | 3 times a wk,20-30 min each time | 12 weeks |
| Luyao Jiang2024 | BA | LI15,LI14,LI11,SJ5,LI4,EX-UE9,LI4 | Deqi sensation,Lifting and Thrusting Technique | 5 times a wk,30 min each time | 4 weeks |
| Minhui Chen2023 | SA | Parietal midline, healthy parietal anterior temporal oblique, parietal 2 lines | Twirling | 5 times a wk,30 min each time | 4 weeks |
| Mingzhu Xu2023 | BA | EX-LE4,ST35,EX-LE2,ST32,ST36,ST36,LR3,SP9,SP6 | Twirling | 5 times a wk,30 min each time | 4 weeks |
| Shaoyang Cui2015 | BA | EX-LE4,ST35,EX-LE2,ST32,ST36,ST36,LR3,SP9,SP6 | Twirling | 5 times a wk,30 min each time | 4 weeks |
| Rui Zhang2017 | BA | GV20，Parietal anterior temporal oblique on the side of the lesion | Deqi sensation, twirling | 6 times a wk,20 min each time | 4 weeks |
| Xiping Zhang2018 | BA | LI10-LI15,LI4,SJ11-SJ14,SJ5,SJ6 | Catgut Embedding Therapy | every two weeks | 6 weeks |
| Hangfan Zhou2017 | BA | LU5,LI11,PC6,LI13,LI15,SI9,SJ10,LI4,SJ5 ,SJ4 | Twirling | 5 times a wk,30 min each time | 12 weeks |
| Keyong Cao2022 | BA | ST36,ST40 ,GB39,LR3 ,KI3 | Deqi sensation, twirling | 5 times a wk,20 min each time | 6 weeks |
| Jun Hu2022 | BA+ES | Choose points such as ST36, GB34, etc. depending on the condition. | 1 -100 Hz, pulse width 0.3 ms | 10 times a wk,15 min each time | 4 weeks |
| Di Zhu2019 | SA | Parietal temporal anteroposterior oblique line on the side of the lesion on the line from GV20 to the temporal region | Twirling | 2 times/day, every other day,30 min each time | 8 weeks |
| Xinfang Sun2015 | BA | Liv3,St40,St36,Gb34,Ki7,Liv2,Li4 | Twirling | 7 times a wk,30 min each time | 4 weeks |
| Su Zheng2018 | BA+ES | Gb34,Sj5,Li4,Li15,Liv2,Pc6,Sj14,Li11,Sj16,Li5,Li6,St8 | Twirling,2-4HZ | 6 times a wk,20 min each time | 6 weeks |
| Xiaoli Song2024 | BA | Pc6,Li4,Liv2,Gb34,St36,Sp6 | Deqi sensation, twirling | 5 times a wk,30 min each time | 8 weeks |
| Daojin Xia2021 | ES | Li15,Liv2,LI10,SJ5 | 100 Hz, pulse width 200 μs, maximum stimulus intensity 100 mA | 6 times a wk,20 min each time | 4 weeks |
| Zhi Tan2020 | SA | Anterior parietotemporal oblique, posterior parietotemporal oblique | Deqi sensation, twirling | 12 times a wk,45 min each time | 6 weeks |
| Lixia Chen2022 | SA | Parietal temporal anteroposterior oblique line on the side of the lesion on the line from GV20 to the temporal region | Twirling | 5 times a wk,30 min each time | 8 weeks |
| Jianming Li2022 | SA | Du20,Du22,Du24,Du25,Du26,Gb1,Du11 | Twirling | 6 times a wk,6 h each time | 6 weeks |
| Zhenyu Ma 2019 | SA | Parietal midline, parietal anterior temporal slope and parietal posterior temporal slope | Twirling | 6 times a wk | 4 weeks |
| Xiangping Zhao2022 | BA | LI15,Liv2,LI10,SJ5,LI4 | Deqi sensation, twirling | 6 times a wk,30min each time | 4 weeks |
| Xinting Wang2021 | SA | Anterior parietotemporal oblique, posterior parietotemporal oblique | Twirling | 5 times a wk,40 min each time | 4 weeks |
| Aijun Wang2022 | SA | Parietal temporal anteroposterior oblique and parietal midline region | Twirling | 5 times a wk,40 min each time | 8 weeks |
| Sheng Ge2021 | SA | Parietal temporal anteroposterior oblique and parietal midline region | Twirling | 5 times a wk,40 min each time | 4 weeks |
| Lei Pang2022 | SA | Parietal temporal anteroposterior oblique line on the side of the lesion on the line from GV20 to the temporal region | Twirling | 2 times/day, every other day,20 min each time | 12 weeks |
| Lixia Chen2021 | SA | Parietal temporal anteroposterior oblique and parietal midline on the side of the lesion | Twirling | 10 times a wk,40 min each time | 8 weeks |
| Qiang He2023 | BA | SP6,BL40,PC6, LI11,HT1 | Deqi sensation,moxibustion | 5 times a wk,30 min each time | 4 weeks |
| Pingping Gou2023 | BA | LI10,LI15,LI14,TE14,BL17,LI8,LI11,LI4,TE5,EX-UE9 | Deqi sensation, twirling,moxibustion | 5 times a wk | 4 weeks |
| Qiong Luo2020 | BA | BL1,GB21,LU5,TE15,LI4,BL17,ST32,ST34,SP9,SP6 | Deqi sensation,moxibustion | 5 times a wk | 4 weeks |
| Yanan Zhang2021 | BA | EX-UE9 | Deqi sensation,Twirling | 6 times a wk,30 min each time | 4 weeks |
| Can Duan2020 | BA | KI3,LR3,UB25,PC6,SP6,HT1,LU5,ST36,LI15,LI4 | Deqi sensation,Twirling | 5 times a wk,20 min each time | 4 weeks |
| Yanan Zhang2020 | BA | GV20,TE17,LI11,PC6,LI4,ST36,SP6,LR3 | Deqi sensation,Twirling | 5 times a wk,30 min each time | 4 weeks |
| Jinjing Zhang2024 | BA | Anterior parietotemporal oblique, posterior parietotemporal oblique,LI15,LI11,LI10,TE5, LI4 | Twirling | 6 times a wk,30 min each time | 2 weeks |
| Han Liu2019 | BA | front part of the parietal temporal slope,LI10,LI4,LI11,LI5,KI19 | Twirling | 5 times a wk,30 min each time | 4 weeks |
| Ning Zhang2021 | BA | GB21-GB23,LI11,LI4,TE5,ST36,SP6,LR3 | Twirling | 6 times a wk,30 min each time | 6weeks |
| Shaoyang Cui2017 | BA | Groin area, depression below the mandible, depression above the laryngeal node,ST32,LR3,ST36,ST35,LE4,LE2,SP9,SP6,ST4,LI20,CV4,CV3,SP6,ST25,CV4,ST37,GV2,EX-HN5 | Deqi sensation,Twirling | 6 times a wk,30 min each time | 4 weeks |
| Xiuhua Zhang2018 | BA | Inguinal region, depression below the mandible，ST32,LR3,ST36,ST35,EX-LE4,EX-LE2,SP9,SP6,ST4,LI20,CV23 | Deqi sensation,Twirling | 5 times a wk,30 min each time | 4 weeks |
| Qin Yang2020 | BA+ES | GB30,BL36,BL37,SP15,GB31,BL18,ST36,GB34,GB39 | 10mA,100Hz | 5 times a wk,30 min each time | 4 weeks |
| Jingjun Xie2018 | BA | fronto-parietal oblique line,GB39,TE15,GB21,LI11,LI10,LI15,LI4 | Deqi sensation,Twirling | 5 times a wk,30 min each time | 4 weeks |

SA = scalp acupuncture; BA = body acupuncture; ES = electrical stimulation

**eTable 8: Subgroup analysis of the effect of vitamin D on cancer mORtality Subgroup** **title**

**e**

|  | No. of trials | No. of patients | I2 | Risk Ratio, 95%CI | P fOR intera ction |
| --- | --- | --- | --- | --- | --- |
| Overall | 5 | 39197 | 0% | 0.84 [0.74, 0.96] |  |
| No. of patients |  |  |  |  |  |
| ≥2000 | 4 | 38957 | 0% | 0.85 [0.74, 0.97] | 0.93 |
| <2000 | 1 | 230 | NA | 0.97 [0.06, 15.29] |  |
| No. of events |  |  |  |  |  |
| ≥200 | 2 | 31163 | 0% | 0.84 [0.72, 0.97] | 0.69 |
| <200 | 3 | 8034 | 0% | 0.89 [0.68, 1.17] |  |
| Age |  |  |  |  |  |
| ≥70 | 2 | 7978 | 0% | 0.85 [0.72, 1.02] | 0.90 |
| <70 | 3 | 31219 | 0% | 0.84 [0.69, 1.02] |  |
| Sex |  |  |  |  |  |
| Female | 0 | 0 | NA | NA | NA |
| Both | 5 | 39197 | 0% | 0.84 [0.74, 0.96] |  |
| Baseline 25(OH)D (nmol/L) |  |  |  |  |  |
| ≥50 | 2 | 30979 | 0% | 0.84 [0.69, 1.02] | 0.95 |
| <50 | 2 | 5532 | 0% | 0.85 [0.69, 1.04] |  |
| Published year |  |  |  |  |  |
| BefORe 2014 | 2 | 7978 | 0% | 0.85 [0.72, 1.02] | 0.90 |
| In OR after 2014 | 3 | 31219 | 0% | 0.84 [0.69, 1.02] |  |
| Type of vitamin D |  |  |  |  |  |
| Vitamin D3 | 5 | 39197 | 0% | 0.84 [0.74, 0.96] | NA |
| Vitamin D2 | 0 | 0 | NA | NA |  |
| Daily dose equivalent |  |  |  |  |  |
| ≥2000 IU | 3 | 31219 | 0% | 0.84 [0.69, 1.02] | 0.90 |
| <2000 IU | 2 | 7978 | 0% | 0.85 [0.72, 1.02] |  |
| Timing |  |  |  |  |  |
| Daily | 2 | 31163 | 0% | 0.84 [0.72, 0.97] | 0.69 |
| Intermittently | 3 | 8034 | 0% | 0.89 [0.68, 1.17] |  |
| Residential status |  |  |  |  |  |
| Community | 5 | 39197 | 0% | 0.84 [0.74, 0.96] | NA |
| Institution | 0 | 0 | NA | NA |  |
| Follow-up |  |  |  |  |  |
| Follow-up ≥3 y | 4 | 39197 | 0% | 0.85 [0.74, 0.97] | 0.93 |
| Follow-up <3 y | 1 | 240 | NA | 0.97 [0.06, 15.29] |  |

**eFigure 1: Risk of bias summary: review authORs' judgements about each risk**

**of bias item fOR each included study.**


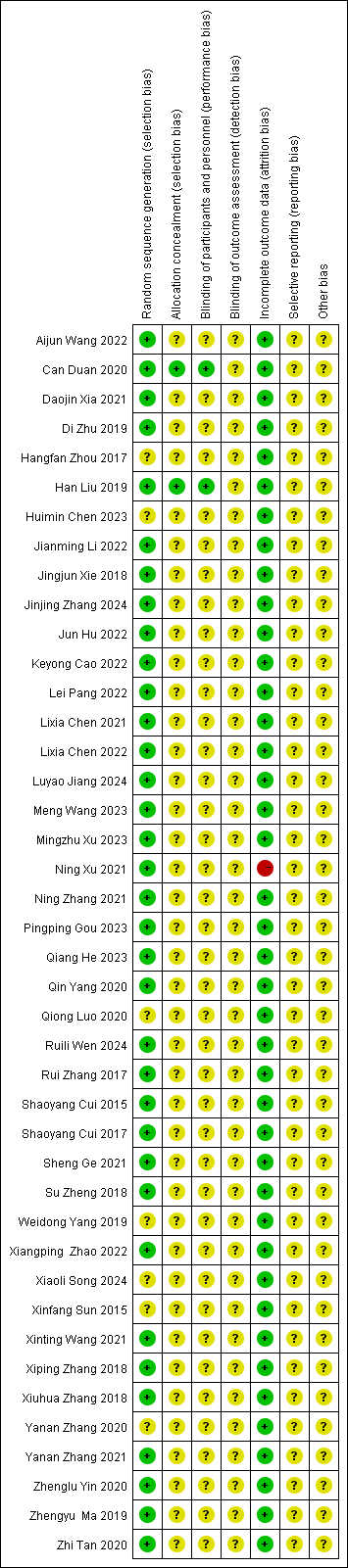


**eFigure 2: Risk of bias graph: review authORs' judgements about each risk of**

**bias item presented as percentages across all included studies**


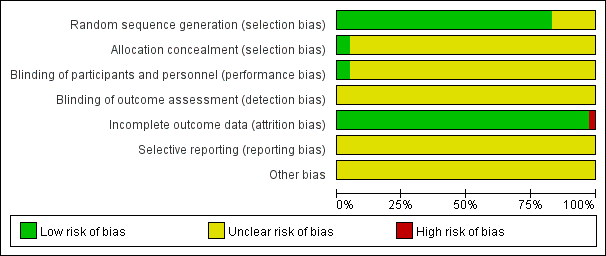


**eFigure 3: Funnel plot of FMA-T**

**
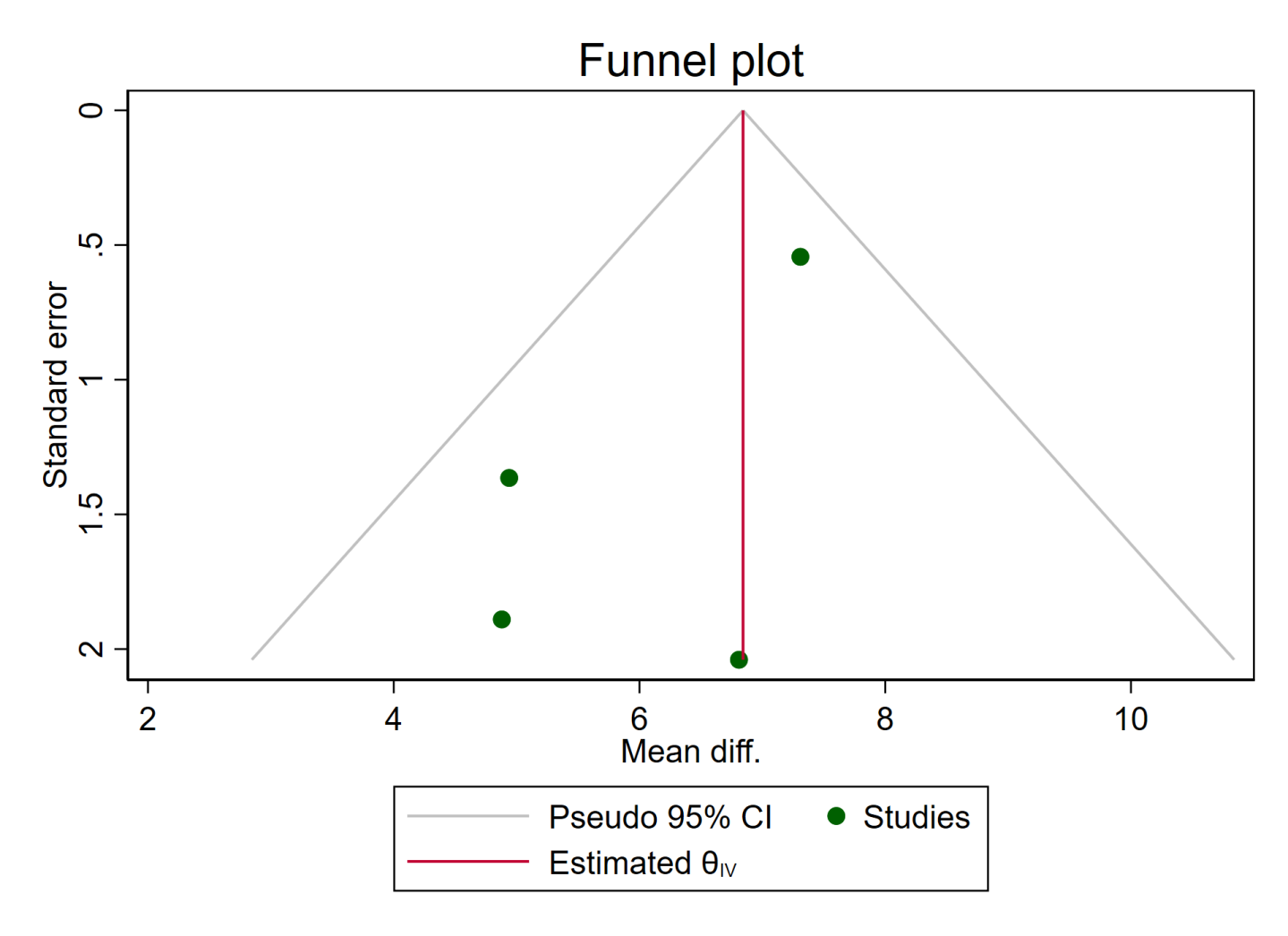
**

**eFigure 4: Funnel plot of FMA-UE**

**
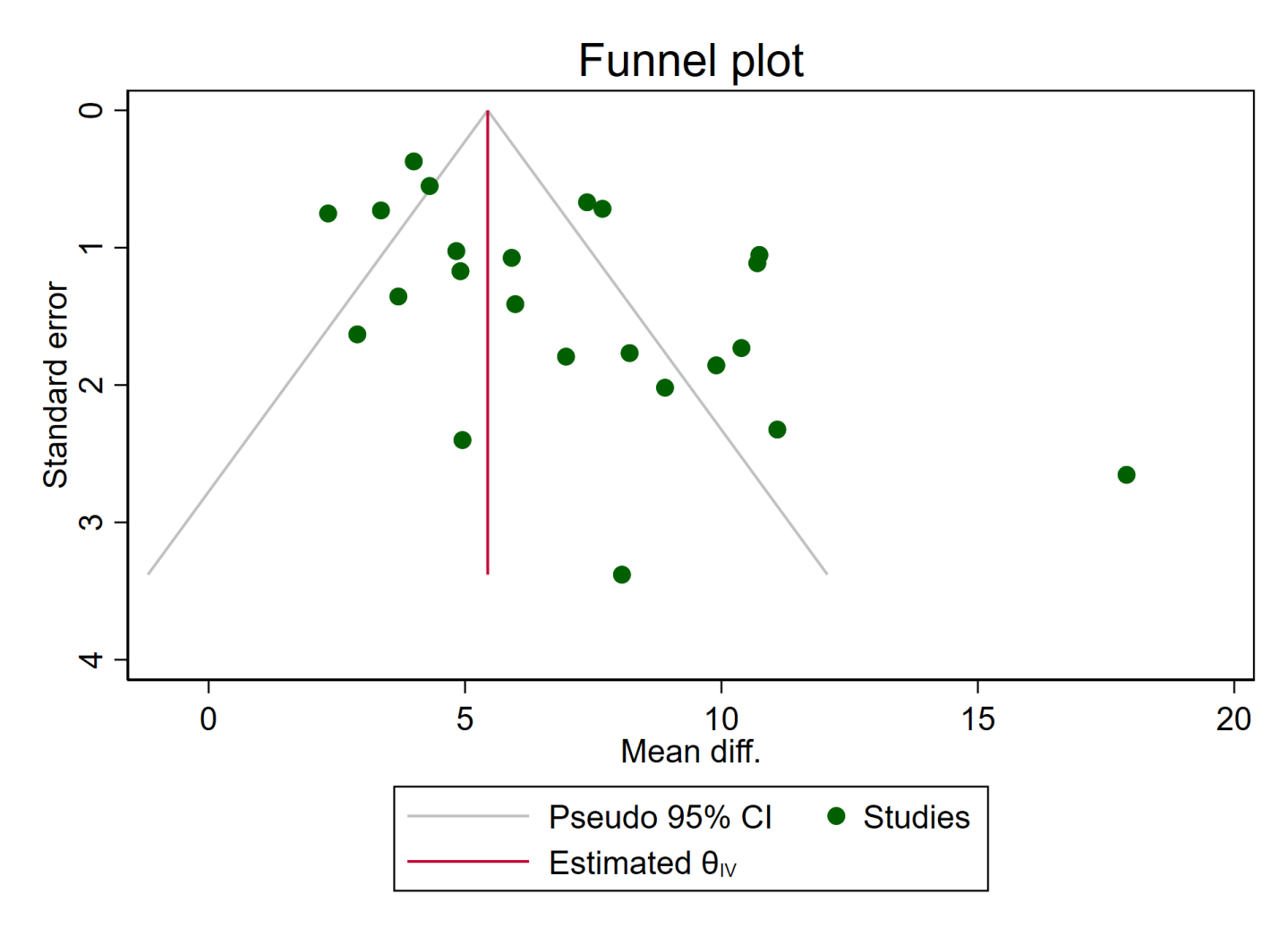
**

**eFigure 5: Funnel plot of FMA-L**

**
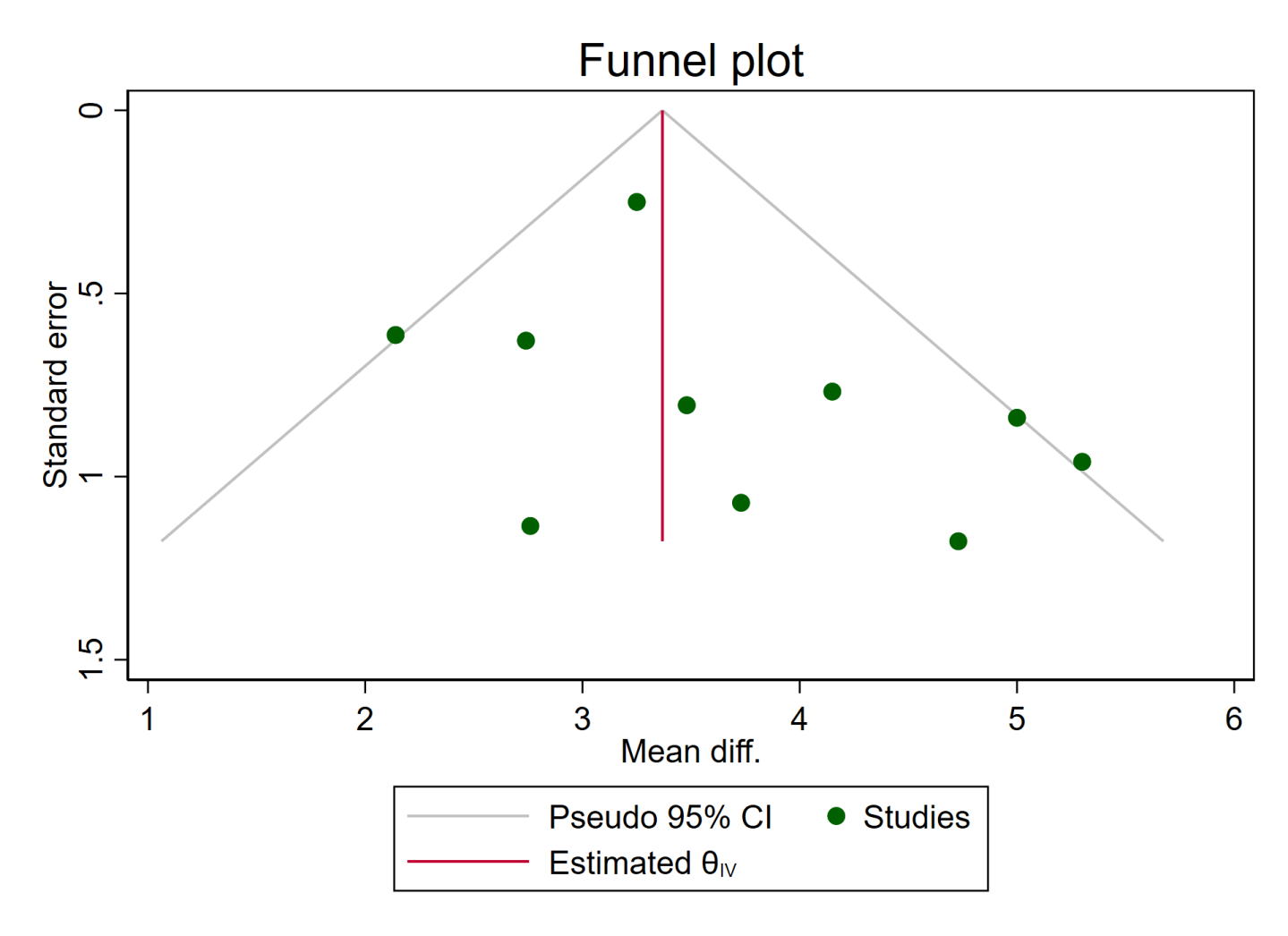
**

**eFigure 6: FMA-T sensitivity analysis**


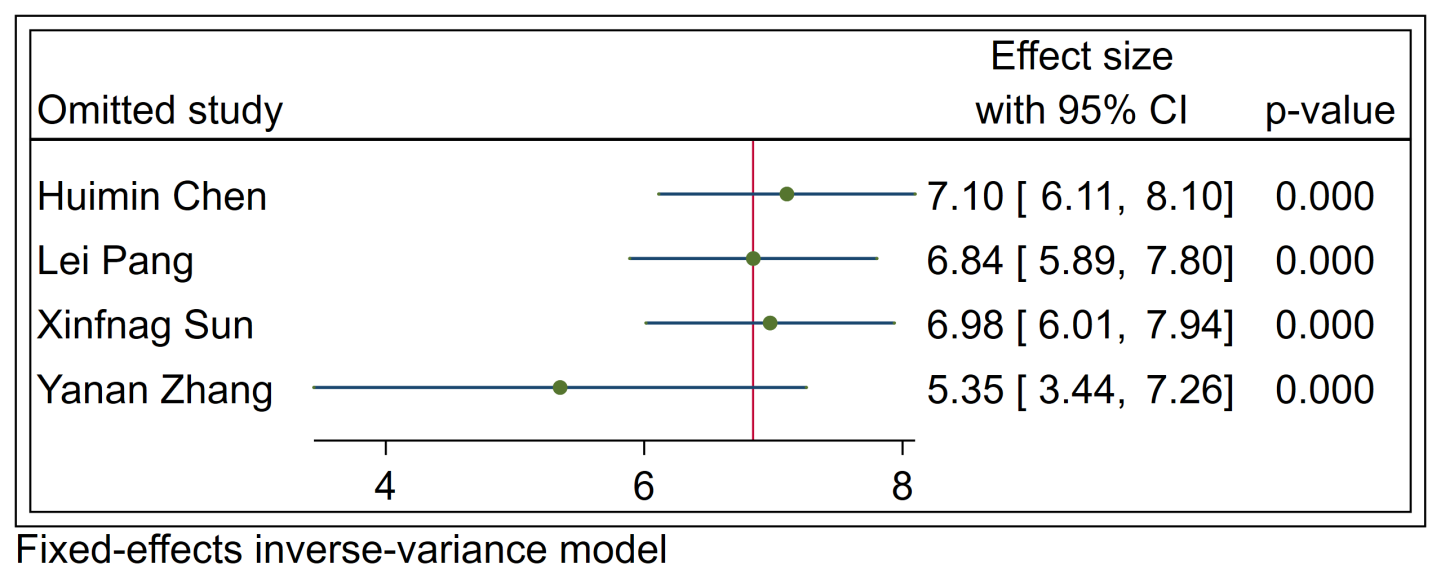


**eFigure 7: FMA-UE sensitivity analysis**


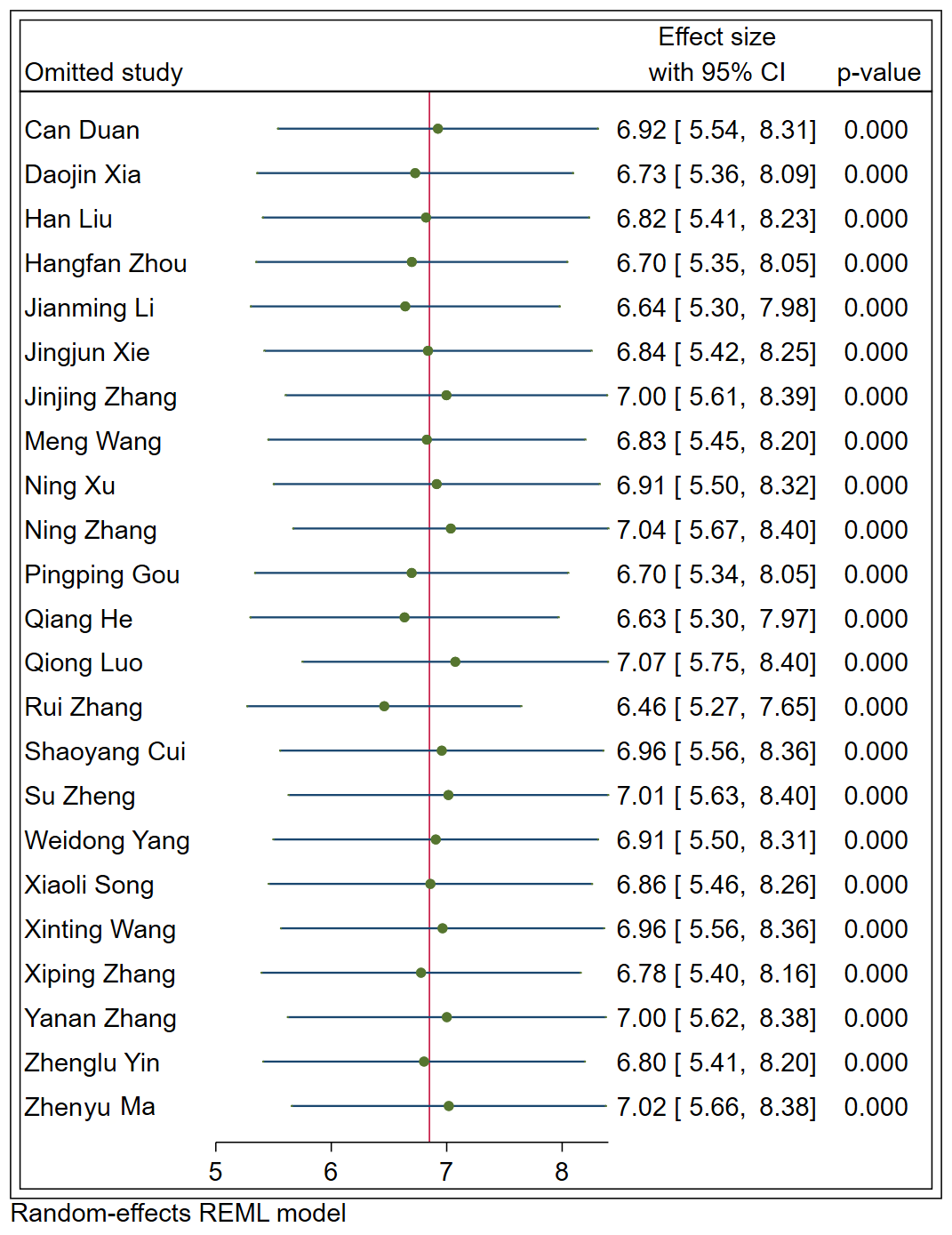


**eFigure 8: FMA-L sensitivity analysis**


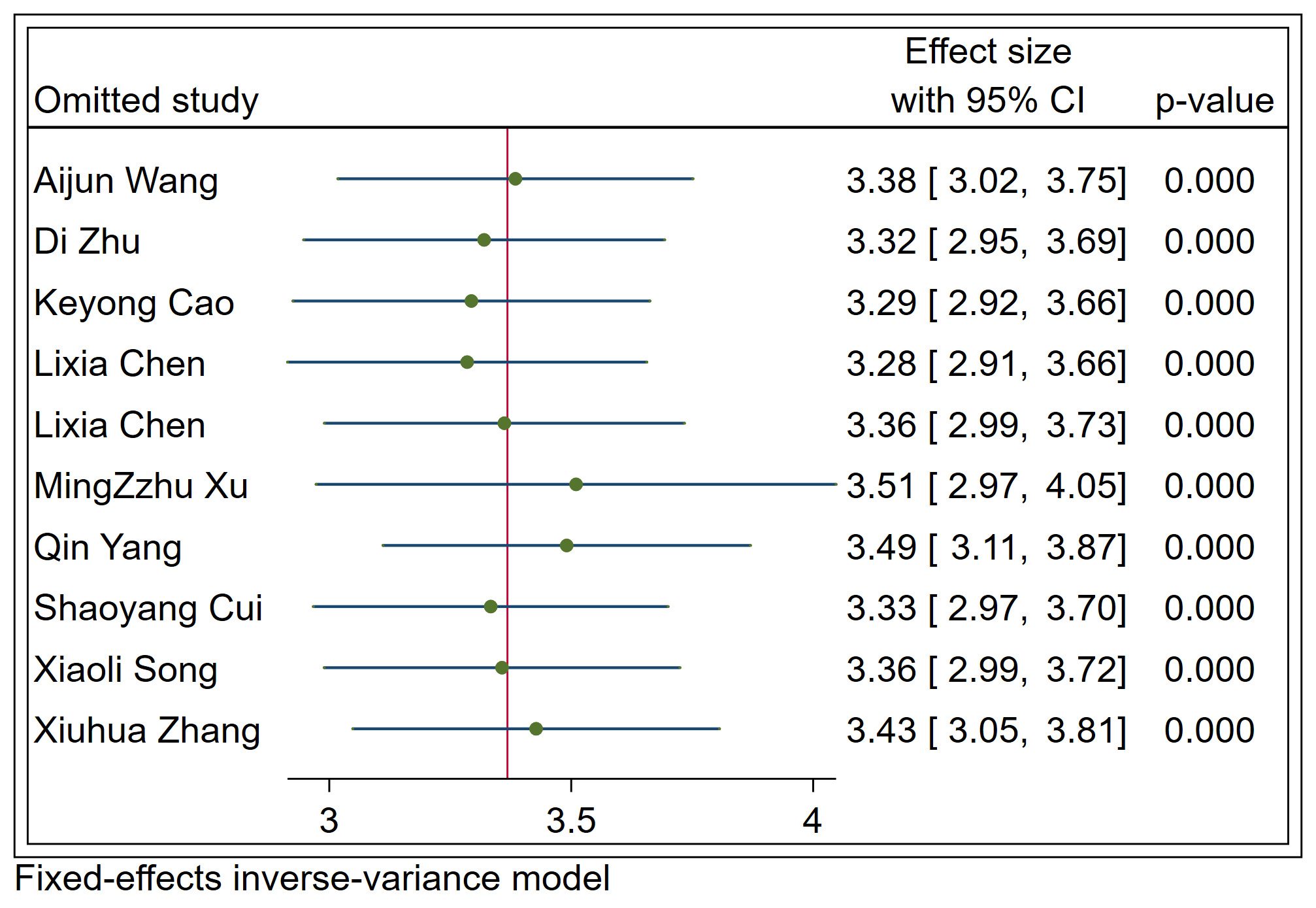


**eFigure 9: Funnel plot of MBI**


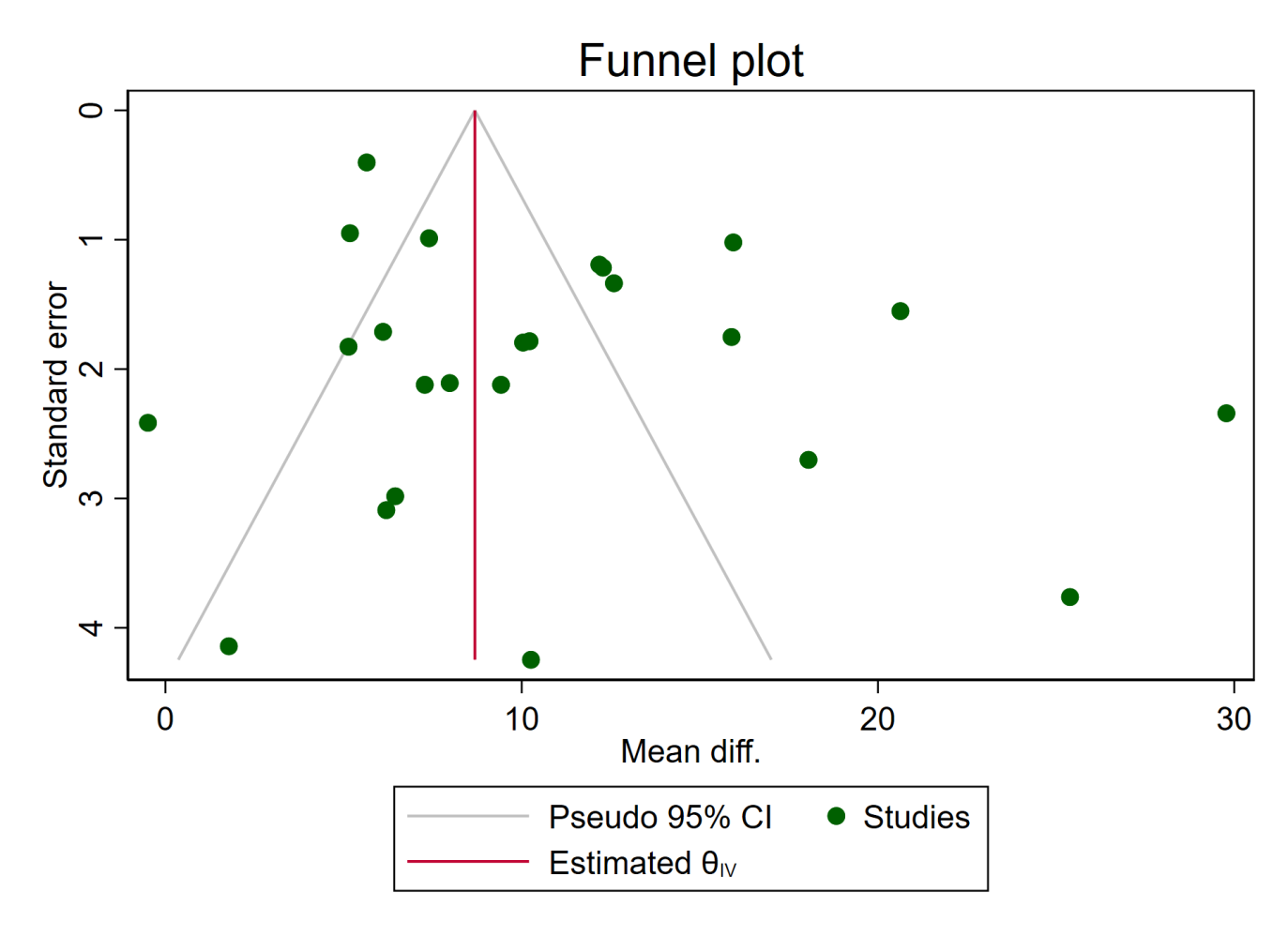


**eFigure 10: Funnel plot of MAS**

**
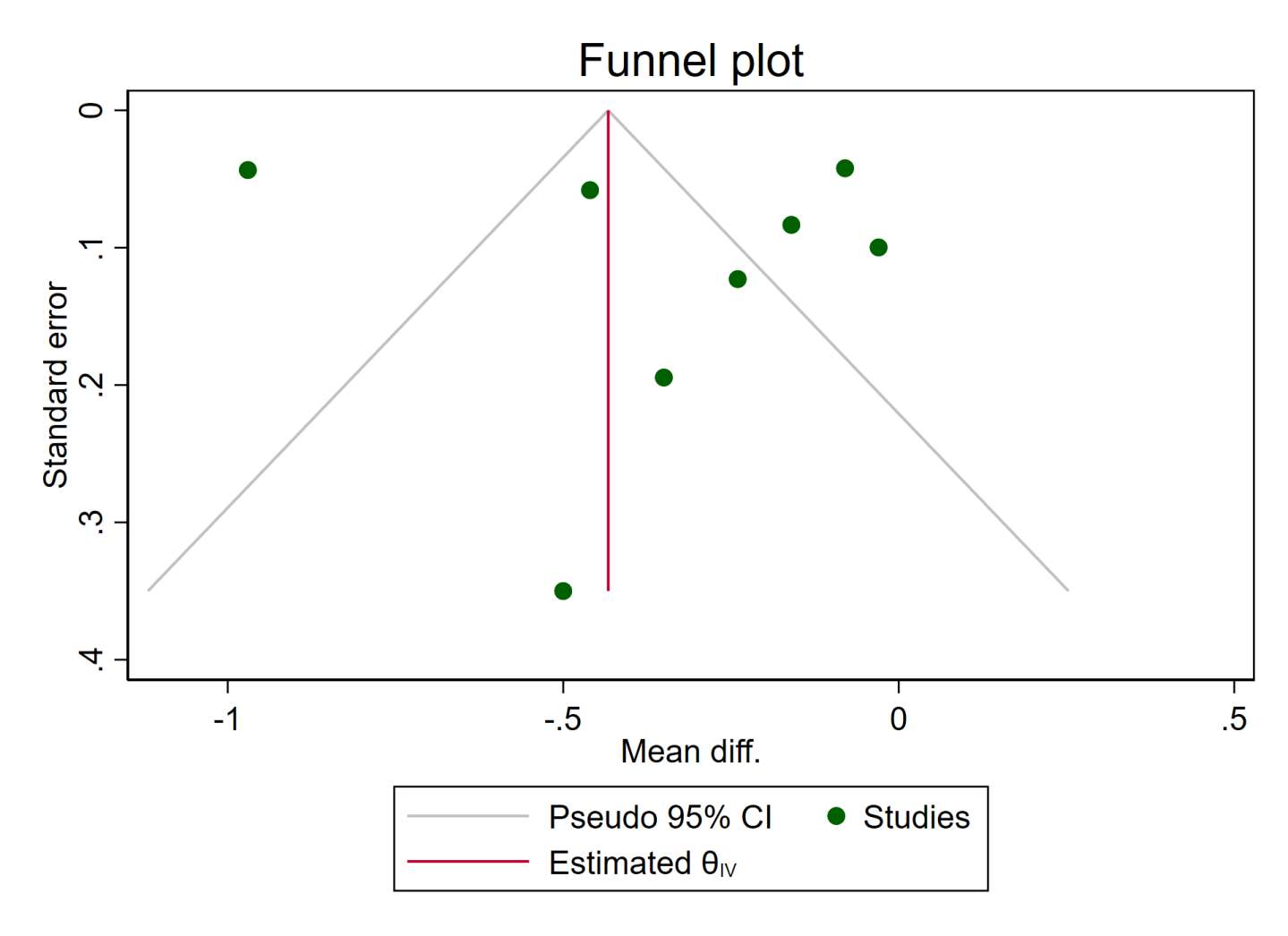
**

**eFigure 11: Funnel plot of Total effective rate**


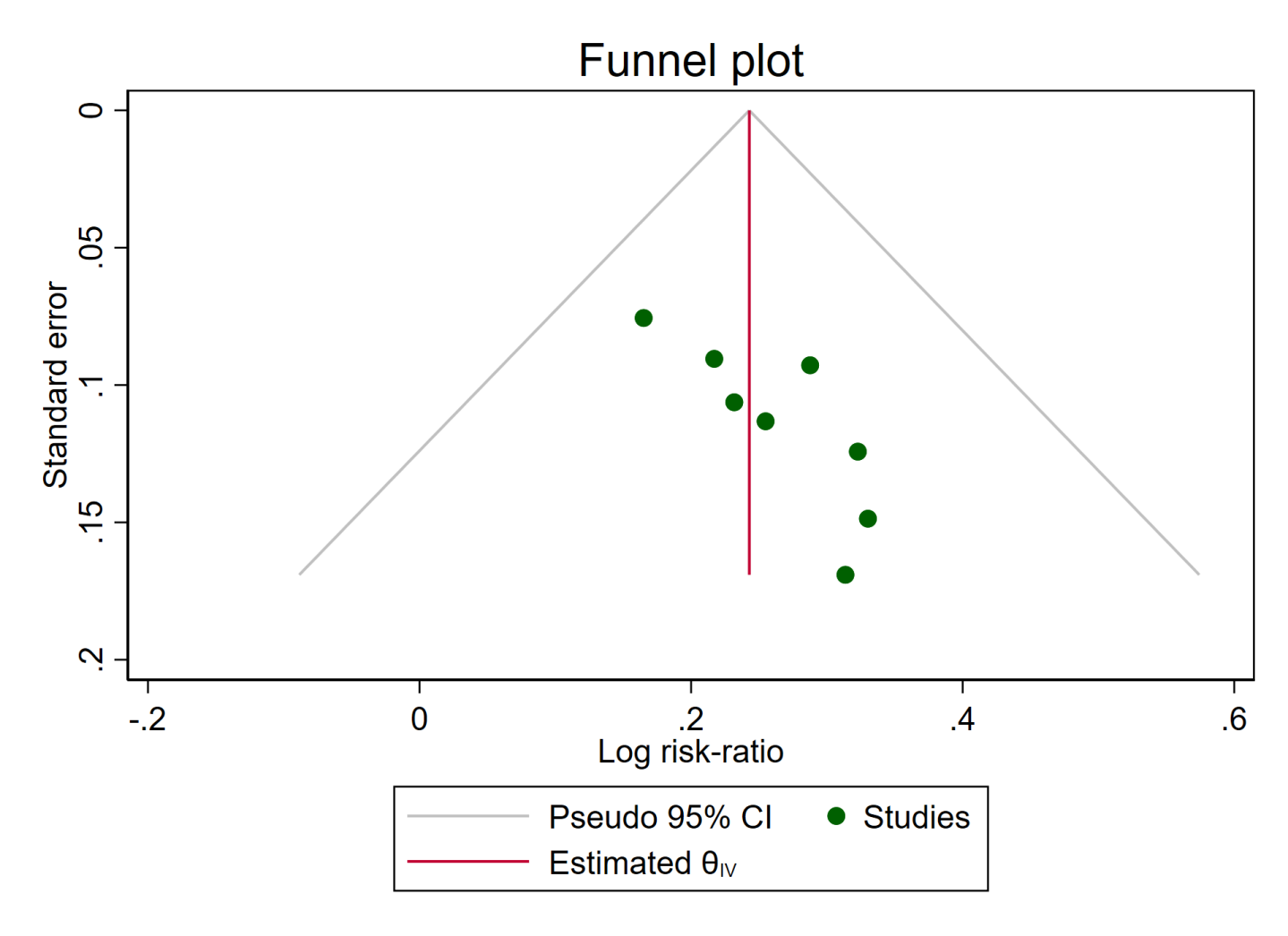


**eFigure 12: MBI sensitivity analysis**


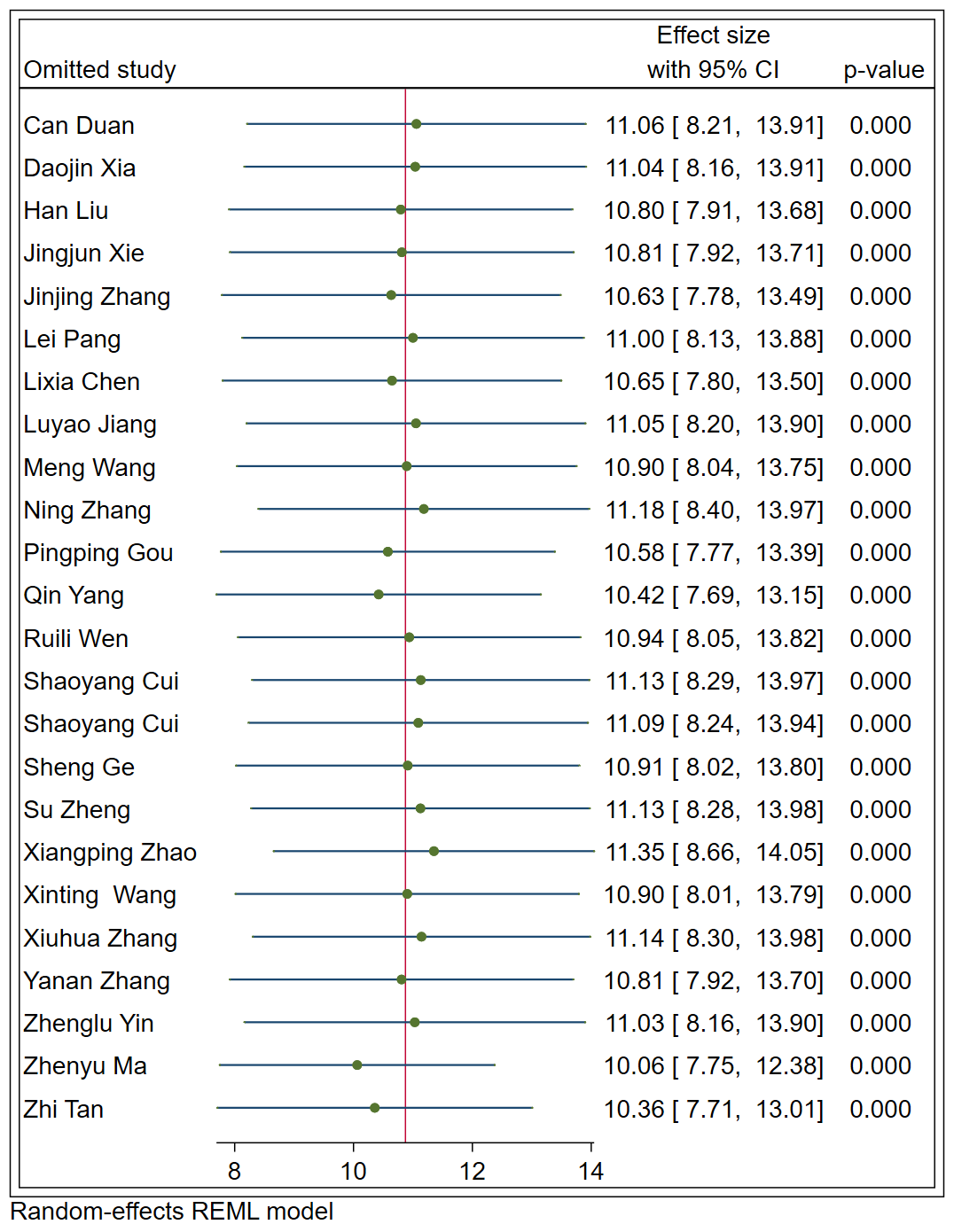


**eFigure 13: MAS sensitivity analysis**


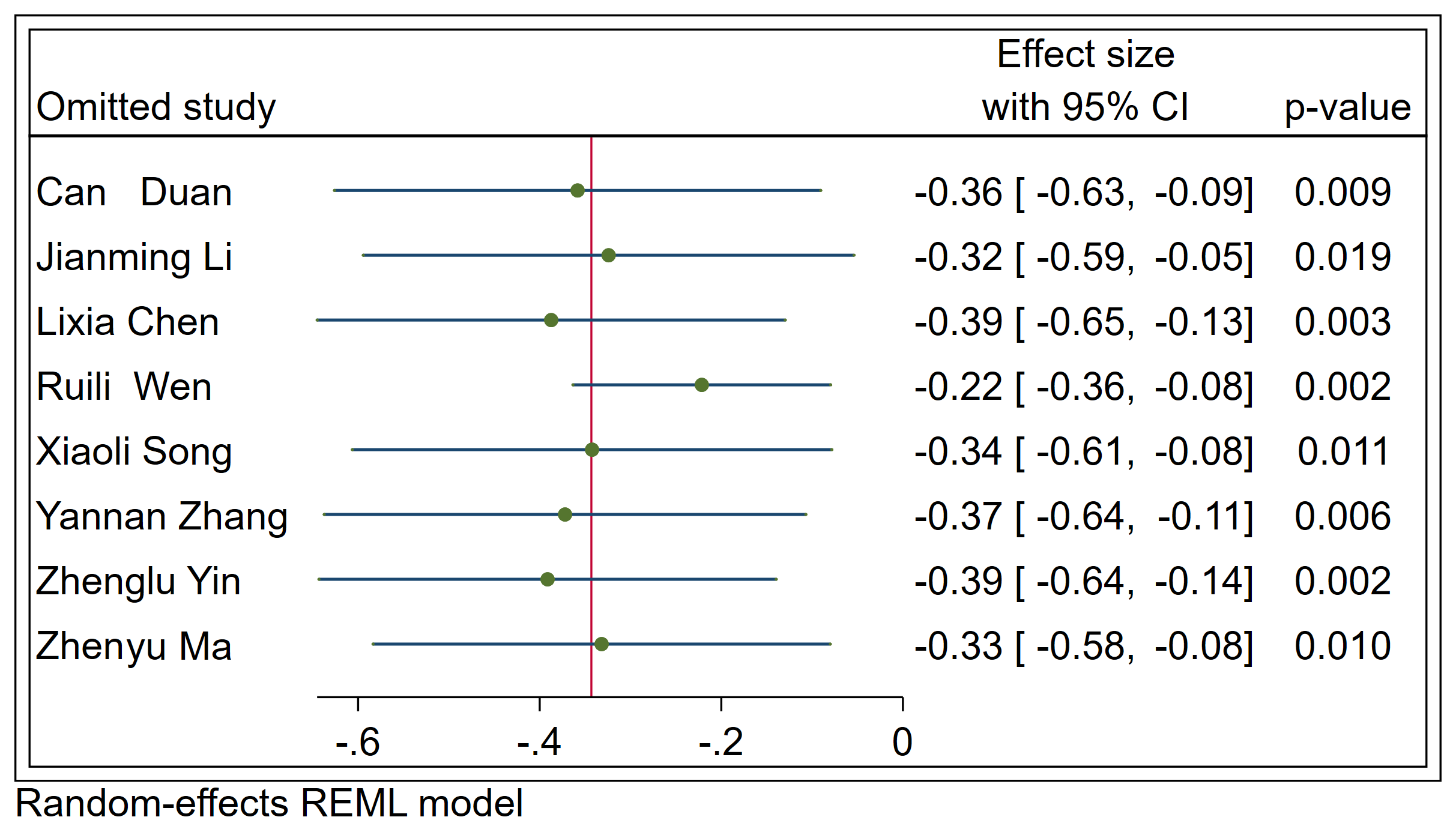


**eFigure 14: Total effective rate sensitivity analysis**

**
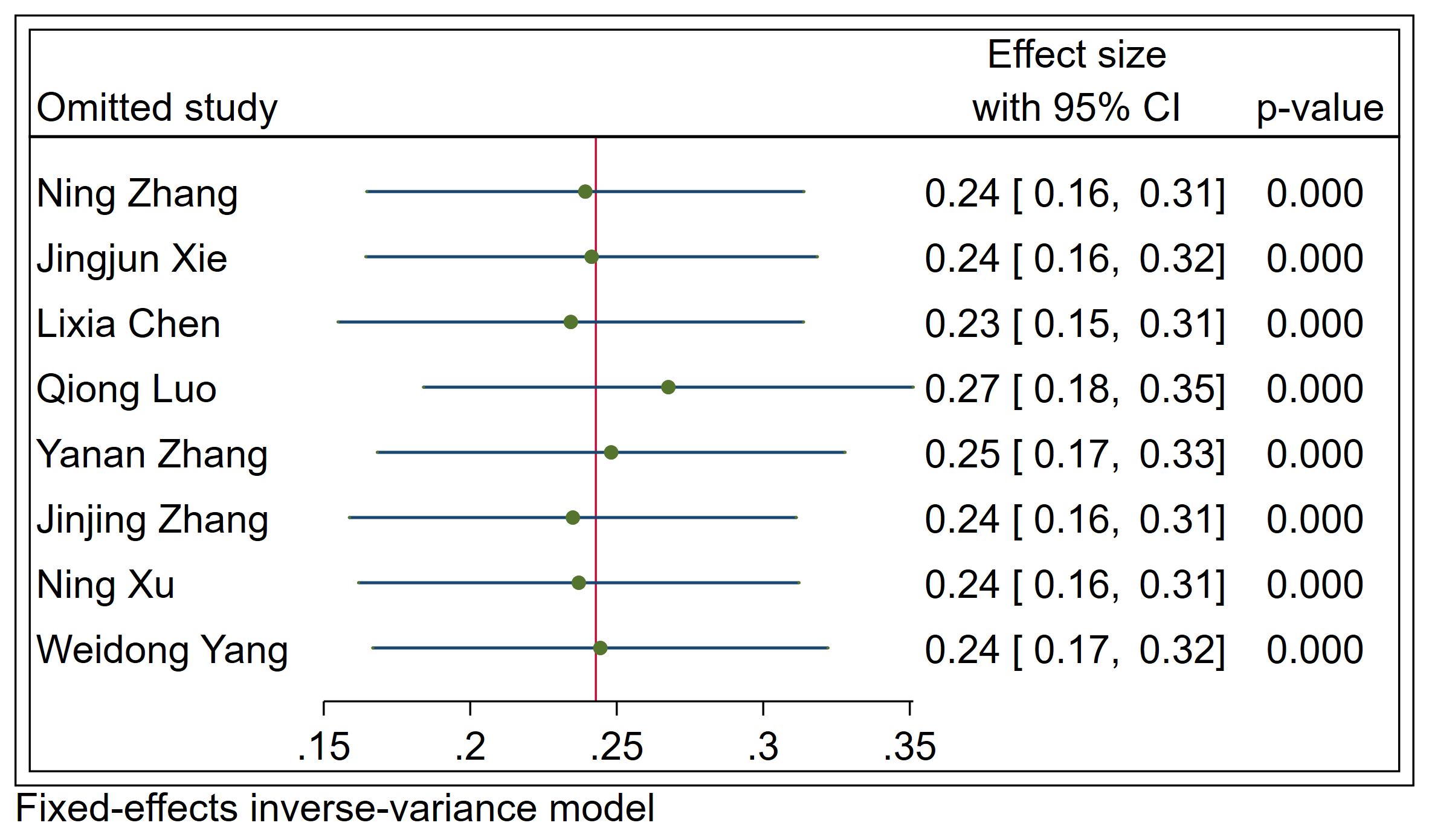
**
